# Mechanistic Study of Carbon Dioxide Hydrogenation over Pd/ZnO‐Based Catalysts: The Role of Palladium–Zinc Alloy in Selective Methanol Synthesis

**DOI:** 10.1002/anie.202103087

**Published:** 2021-06-24

**Authors:** Maxim Zabilskiy, Vitaly L. Sushkevich, Mark A. Newton, Frank Krumeich, Maarten Nachtegaal, Jeroen A. van Bokhoven

**Affiliations:** ^1^ Laboratory for Catalysis and Sustainable Chemistry Paul Scherrer Institute 5232 Villigen Switzerland; ^2^ Institute for Chemistry and Bioengineering ETH Zurich Vladimir-Prelog-Weg 1 8093 Zürich Switzerland

**Keywords:** CO_2_ hydrogenation, isotope labeling, methanol, operando XAS, PdZn alloy

## Abstract

Pd/ZnO catalysts show good activity and high selectivity to methanol during catalytic CO_2_ hydrogenation. The Pd‐Zn alloy phase has usually been considered as the active phase, though mechanistic studies under operando conditions have not been conducted to verify this. Here, we report a mechanistic study under realistic conditions of methanol synthesis, using in situ and operando X‐ray absorption spectroscopy, X‐ray powder diffraction, and time‐resolved isotope labeling experiments coupled with FTIR spectroscopy and mass spectrometry. Pd‐Zn alloy‐based catalysts, prepared through reduction of a heterobimetallic Pd^II^Zn^II^ acetate bridge complex, and which do not contain zinc oxide or any PdZn/ZnO interface, produce mostly CO. The Pd‐Zn phase is associated with the formation of CO, and does not provide the active sites required to produce methanol from the direct hydrogenation of carbon dioxide. The presence of a ZnO phase, in contact with a Pd‐Zn phase, is essential for efficient methanol production.

## Graphical abstract

In this operando study of methanol synthesis over Pd/ZnO catalysts, we show that the palladium–zinc alloy phase by itself is not particularly good at selectively hydrogenating carbon dioxide. Instead, and in the absence of a co‐existing zinc oxide phase, the palladium–zinc alloy primarily yields carbon monoxide. When zinc oxide and palladium–zinc alloy nanoparticles are both present, the system becomes a far more active and selective catalyst.

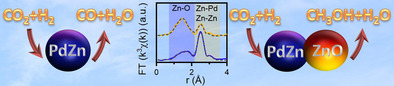

## Introduction

Despite decades of effort, a commercially viable synthesis of methanol from carbon dioxide, has yet to be realised, and the development of an appropriate catalyst formulation is still very much in progress. The commercial catalyst for methanol synthesis from syngas (Cu/ZnO/Al_2_O_3_), as well as other copper‐based materials, such as Cu/ZrO_2_, Cu/CeO_2_, Cu/ZnO/ZrO_2_ and Cu/Mo_2_C, are often the first choice for studying carbon dioxide hydrogenation to methanol. However, limited activity and poor selectivity to methanol due to the reverse water gas shift reaction (RWGS) have forced the investigation of alternative catalyst compositions, for instance: In_2_O_3_ based materials;[^1^, ^2^, ^3^, ^4^] palladium‐containing catalysts;[^5^, ^6^, ^7^] and gallium‐based intermetallic compounds.[^8^, ^9^, ^10^] Among these Pd/ZnO catalysts, which show good activity and high selectivity to methanol, are of a great scientific interest.[^11^, ^12^, ^13^, ^14^, ^15^]

In order to improve the performance of catalysts for methanol synthesis from carbon dioxide, an understanding of the reaction mechanism, and the structure of the active sites, is of a considerable importance. Under a hydrogen rich atmosphere and at elevated temperature (≥150 °C), Pd/ZnO is known to form a palladium–zinc alloy phase, which is considered as providing the active sites for various catalytic applications including methanol steam reforming, reverse water gas shift, and hydrogenation reactions.[^6^, ^16^, ^17^, ^18^, ^19^, ^20^, ^21^] The formation of a palladium–zinc alloy phase is also often associated with high activity and selectivity towards methanol in the hydrogenation of carbon dioxide.[^5^, ^15^, ^22^, ^23^] Although the mechanism of methanol synthesis over Pd/ZnO is not fully understood, it is considered that the palladium–zinc alloy is able to generate and stabilize formate intermediate species, which may then be hydrogenated to yield methanol.^[23]^ The association of the palladium–zinc alloy phase with the active sites has, in most studies, solely been based on the fact that this phase is formed during the reductive pre‐treatment of the catalyst. However, in situ and/or operando studies, conducted under reaction relevant conditions (200–260 °C, *P*≥10 bar), which could verify this supposition, have yet to appear. Gentzen et al.,^[24]^ investigated the behavior of a Pd/ZnO system under operando conditions and observed formation of palladium–zinc alloy during dimethyl ether synthesis from carbon monoxide‐rich gas mixtures having a carbon monoxide/hydrogen ratio 1:1, however, methanol synthesis typically utilizes a significantly more reducing feedstock (H_2_/CO_2_=3).

Taking into account recent works,[^25^, ^26^, ^27^, ^28^] which have questioned the role of copper‐zinc alloy in methanol synthesis from carbon dioxide, an operando investigation of carbon dioxide hydrogenation over Pd/ZnO system is warranted to understand the true nature of the active sites and to verify the role that a palladium–zinc alloy phase may have in facilitating this reaction. We therefore report a combined high‐pressure operando study of methanol synthesis over Pd/ZnO catalysts using XAS, XRD and FTIR techniques. We find that, despite the fact that a palladium–zinc alloy phase is present during reaction, methanol is not formed when the support is an inert oxide (e.g. Al_2_O_3_, SiO_2_). Instead, we demonstrate that the co‐existence of zinc oxide with palladium or palladium–zinc phases is required to form methanol with high activity and selectivity in this industrially relevant reaction.

## Results and Discussion

**Figure 1:**
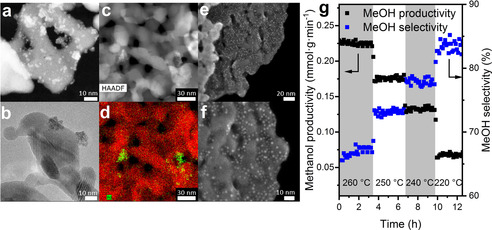
HAADF‐STEM (a) and BF‐STEM (b) of 2Pd‐ZnO‐i. HAADF‐STEM image (c) and corresponding EDX elemental mapping (d) of 2Pd‐ZnO‐i (Zn red, Pd green). SE‐STEM images of 2Pd‐ZnO‐np (e and f). Results of catalytic carbon dioxide hydrogenation (CO_2_:H_2_=1:3) over 2Pd‐ZnO‐np at 50 bar total pressure and WHSV=120 L g^−1^ h^−1^ (g).

Figure 1 shows electron microscopy results of Pd/ZnO after calcination in a flow of air at 400 °C. The 2Pd‐ZnO‐i sample, prepared by impregnation of palladium nitrate solutions, contains unevenly distributed palladium nanoparticles with sizes ranging from 1 to 7 nm (Figure 1 a–d). The sample synthesized using colloidal palladium shows nicely distributed, and essentially monodisperse, palladium nanoparticles with a mean size of 2±0.2 nm (Figure 1 e,f).

Catalytic carbon dioxide hydrogenation over 2Pd‐ZnO‐np at total pressure of 50 bar results in a high selectivity to methanol of between 65 and 85 %, and with carbon monoxide as the sole by‐product (Figure 1 g). Decreasing the reaction temperature from 260 °C to 220 °C yields an even higher methanol selectivity since, at lower temperature, the methanol synthesis reaction increasingly dominates the catalytic hydrogenation of carbon dioxide. The absolute values of methanol productivity are among the highest for Pd/ZnO‐based catalysts thus far reported for this system (Supporting Information, Table S1). In an attempt to understand the fundamental reasons of such a high activity of palladium–zinc catalyst, we have focused our attention on 2Pd‐ZnO‐np sample and used it as the main object in our operando experiments.

**Figure 2:**
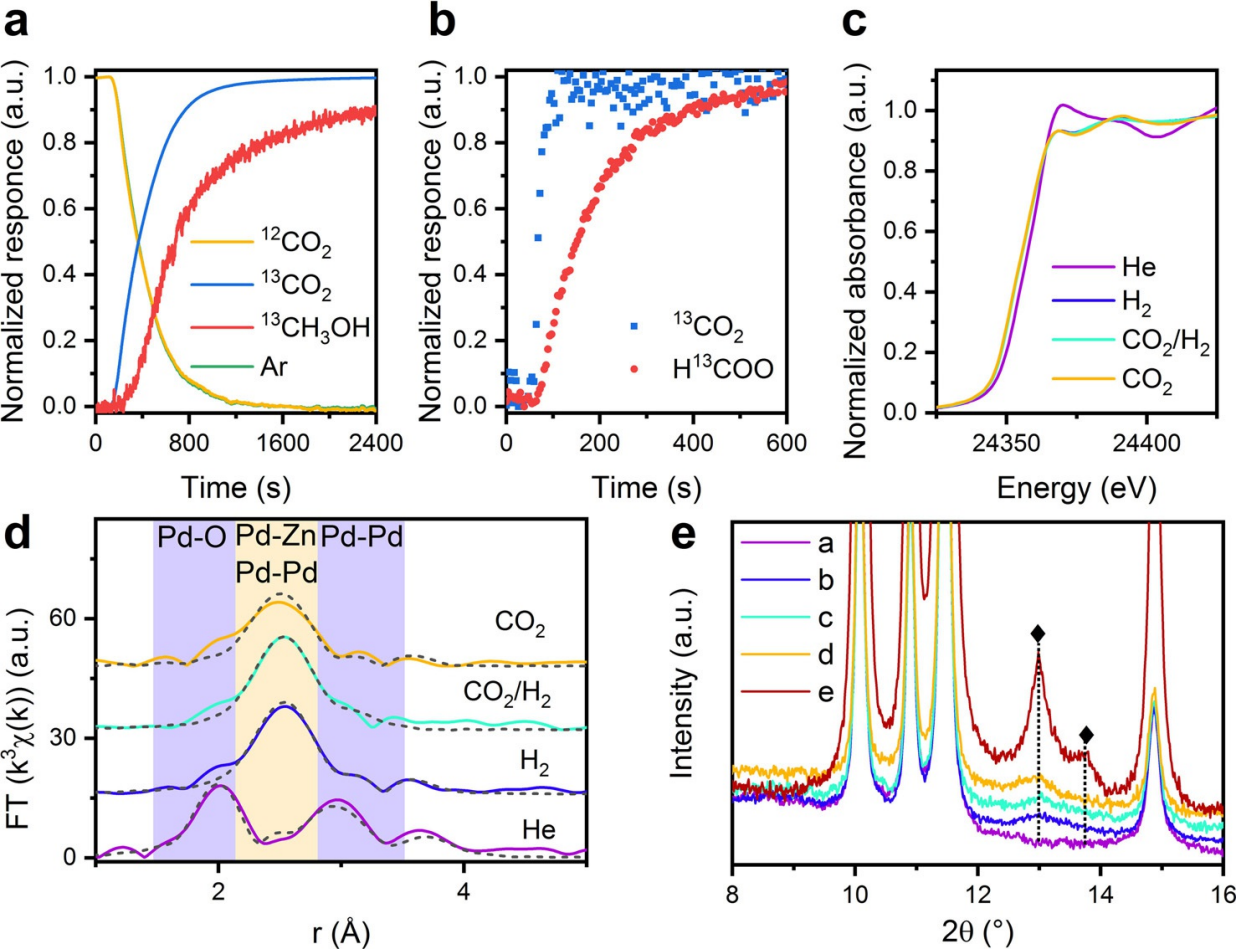
Normalized isotopic transient response curves following the switch from a ^12^CO_2_/H_2_ to ^13^CO_2_/H_2_ mixture during catalytic carbon dioxide hydrogenation over 2Pd‐ZnO‐np at 260 °C and 15 bar total pressure measured by mass‐spectrometry (a) and FTIR spectroscopy (b). Pd K‐edge XANES spectra (c), the corresponding phase corrected Fourier transform representation of the k^3^‐weighted EXAFS (d) and XRD (e) collected during activation and catalytic carbon dioxide hydrogenation over 2Pd‐ZnO‐np catalyst at 260 °C and 15 bar pressure under different sets of conditions (as indicated): under a helium flow (violet); under flowing hydrogen (blue); at steady state under a catalytic CO_2_/H_2_ reaction mixture (aquamarine); and under a flow of carbon dioxide (orange). In each case the solid lines concern the experimental data, the black dotted lines, fits to that data derived from analysis in EXCURV.^[29]^ Diffraction peaks of the palladium–zinc alloy phase present in 2Pd‐ZnO‐np and 2Pd‐ZnO‐i (red XRD pattern) samples are labeled with diamond symbol (⧫).

The kinetic evolution of the products and intermediate species was followed by IR spectroscopy and MS. Figure 2 a shows the normalized MS responses on 2Pd‐ZnO‐np for ^13^C‐labelled and unlabeled carbon dioxide and methanol versus the signal of the tracer (argon), obtained during the isotope switch from ^12^CO_2_/H_2_ to ^13^CO_2_/H_2_. The normalized response of unlabeled carbon dioxide follows the response of the inert tracer, which points to the irreversible activation of carbon dioxide to form the reaction products. This observation is in line with the infrared spectra, which show no bands due to possible intermediates such as carbonate‐like species (Figures S1,S2). In contrast to carbon dioxide, the ^13^CH_3_OH response shows a significant delay with respect to the tracer. This points to the presence of sequential reactions involved in the conversion of carbon dioxide to methanol, along with possible re‐adsorption of methanol on the surface of the catalyst. Infrared spectra, acquired during the reaction of ^12^CO_2_/H_2_ over a 2Pd‐ZnO‐np catalyst, show the presence of the bands centered at 1596 and 1376 cm^−1^ which may be assigned to the asymmetric and symmetric vibrations of bidentate formate species, respectively (Figure S1, Table S2). Our recent study,^[25]^ based on FTIR spectroscopy and XAS analysis, revealed that these formate species correspond to zinc formate species in a copper‐zinc oxide catalyst. No other bands due to methoxy or carbonate species were found in the spectra. Switching of the isotopic composition of the reacting feed from ^12^CO_2_ to ^13^CO_2_ leads to fast exchange of formate species, resulting in a decrease of the intensity of the bands at 1596 and 1376 cm^−1^, which is accompanied by a simultaneous development of bands at 1551 and 1346 cm^−1^ due to ^13^C labeled formates (Figure S2). The difference spectra for both switches show that the exchange is complete and fully reversible. This indicates the involvement of all zinc formate species in the reaction of carbon dioxide hydrogenation to methanol. Moreover, the maximum of the bands does not shift during the isotope exchange, suggesting the presence of a single type of zinc formate species with identical reactivity.

For kinetic analysis, the area of the band at 1596 cm^−1^ due to these formate species and bands due to gas phase carbon dioxide were integrated and compared (Figure 2 b). The signal due to the carbon dioxide matches the behavior of the argon tracer in the MS very closely (Figures 2 a,b). The results clearly show that the kinetic evolution of formate species is significantly delayed with respect to the evolution of the carbon dioxide signal. This unambiguously points to the involvement of formate species to the catalytic cycle of methanol synthesis.

In further experiments, the evolution of both palladium and zinc in the catalyst was followed. A combined operando XAS‐XRD experiment for 2Pd‐ZnO‐np was conducted at 260 °C and 15 bar pressure at the SNBL beamline (BM31, ESRF, France). Figure 2 c shows Pd K‐edge XANES spectra collected during catalyst pre‐treatment and during operando carbon dioxide hydrogenation. After heating in helium to 260 °C, the spectrum of the fresh catalyst resembles well that of the palladium oxide reference (Figures S3,S4). Changing the reaction gas to hydrogen leads to a shift of the white line to lower energy by about 2 eV owing to immediate reduction of palladium oxide and formation of reduced palladium phase (Figure 2 c, Figures S3,S4). According to EXAFS analysis (Figure 2 d, Table S3), XRD (Figure 2 e and Figures S5,S6), and TEM (Figures S7,S8), the resulting phase is a palladium–zinc alloy, in a good agreement with previous studies, where the formation of this phase was observed even at ambient pressure.[^17^, ^21^, ^30^] The formation of the palladium–zinc alloy phase is facilitated by hydrogen spillover and accumulation of hydrogen inside metallic palladium.^[21]^ Furthermore, and irrespective of the difference in palladium particle size and distribution, the 2Pd‐ZnO‐i sample shows very similar behavior during operando XAS study (Figure S3).

In contrast to our previous study,^[25]^ where we have observed changes in Cu K‐edge XANES that result from an oxidative desegregation of zinc from a copper–zinc alloy phase upon switching to a CO_2_/H_2_ mixture, the position of the palladium white line remains constant, not only during carbon dioxide hydrogenation conditions, but even after switching to pure carbon dioxide (Figure 2 c). Since the white line is sensitive to the oxidation state of palladium, this suggests that palladium remains in a metallic state and that the formate species, as observed by IR (Figures S1,S2 and Figure 2 b), are not bound to the palladium. Moreover, the palladium–zinc alloy phase is stable under reaction conditions and under high pressures of carbon dioxide. The Zn K‐edge XANES (Figure S9) also stays constant during transient switches between hydrogen, CO_2_/H_2_ mixture and pure carbon dioxide, for this (2Pd‐ZnO‐np) sample. Taking into account the particle size of the zinc oxide phase, which is in the range 10–30 nm according to TEM and XRD analysis (Figures 1, Figure 2 e and S7b), any surface changes, even if they take place during catalytic reaction, will be difficult to observe using Zn K‐edge XAS. Figure 2 d and Figure S10 show the k^3^‐weighted Pd K‐edge EXAFS derived from the 2Pd‐ZnO‐np sample during the operando XAS experiment. The presence of Pd‐O scattering at 2 Å and two Pd‐Pd scattering shells, at ca. 3.03 and 3.43 Å respectively, are indicative of a nanoscale, PdO structure (Table 1). Therefore, EXAFS yields a picture of 2Pd‐ZnO‐np sample under 15 bar He at 260 °C as being predominantly oxidized in nature, which is consistent with the previously shown XANES (Figure 2 c).

**Table 1:** Structural and statistical parameters derived from the fitting of k^3^‐weighted Pd K‐edge EXAFS for the 2Pd‐ZnO‐np catalyst under different conditions at 260 °C and 15 bar pressure, *K*_min_=2.5, *K*_max_=13, AFAC=0.9.

Conditions	Element	*N* ^[a]^	*r* [Å]^[b]^	DW^[c]^	*E*_F_^[d]^	*R* ^[e]^	Chi^2[f]^
He	O	2.4	2.02	0.008	1.83	28.35	0.55
	Pd	2.3	2.74	0.018			
	Pd	1.2	3.03	0.016			
	Pd	2.9	3.43	0.024			
H_2_	Zn	4.4	2.59	0.019	−6.1 (−6.6)	25.3 (25.7)	0.45 0.43
	Pd	2.2	2.95	0.029			
	Pd (Zn)	0.9	3.51 (3.69)	0.014			
CO_2_/H_2_	Zn	4.4	2.58	0.019	−5.5	45.8	1.25
	Pd	2.1	2.92	0.027			

[a] N=Coordination number. [b] R=bond distance in Angstrom. [c] DW=Debye‐Waller (disorder) factor (2σ^2^) where σ^2^=mean squared displacement of the atom pair with respect to each other. [d] *E*_F_=Fermi energy (eV). [e] R%=Σ_i_^N^ 1/σ_i_ (χ_i_^e^ (*k*)−χ_i_^t^ (*k*))^2^×100 % Where χ_i_^e^ and χ_i_^t^ are the experimental and theoretical EXAFS respectively, and k is the photoelectron wave vector (Å^−1^). σ_i_ is the uncertainty in the data, with 1/σ_i_=k_i_^n^/ Σ_j_^N^ k_i_^n^ (χ_i_^e^ (κ_j_))^2^. [f] Chi^2^=statistical measure of goodness of fit (×10^−6^).

A switch to a hydrogen flow results in significant changes in the FT EXAFS. The peak at 2 Å (due to Pd‐O scattering) disappears, while a new peak at ca. 2.59 Å arises (Figure 2 d). This corresponds to a complete reduction of the palladium present, along with some of the zinc oxide, to yield a highly dispersed palladium–zinc alloy phase (*R*_Pd‐Zn_=2.59 Å, *R*_Pd‐Pd_=2.95 Å).^[21]^ Exposure to the reaction mixture, and then carbon dioxide, does not affect the palladium–zinc alloy (Figure 2 d). Overall, the fitting of k^3^‐weighted Pd K‐edge EXAFS of 2Pd‐ZnO‐np (*R*_Pd‐Zn_=2.59 Å, *R*_Pd‐Pd_=2.92 Å) shows that, once reduced in hydrogen, the nanoparticulate palladium–zinc phase is retained under all of the conditions applied, irrespective of the gaseous environment experienced (Tables 1 and S3).

Figure 2 e shows the XRD patterns of 2Pd‐ZnO‐np collected at the end of every step during the XAS experiment (Figure 2 c and d). The change of gas atmosphere from helium to hydrogen results in the formation of very broad (FWHM≈1) overlapping peaks. For clarity, the XRD pattern of 2Pd‐ZnO‐i (containing larger palladium nanoparticles) collected under hydrogen atmosphere was added to Figure 2 e (other XRD patterns for this sample can be found in Figure S5). The XRD pattern of the spent 2Pd‐ZnO‐np sample shows the (111) diffraction peak of a palladium–zinc alloy phase after catalytic carbon dioxide hydrogenation. Other diffraction peaks present in this pattern, correspond to the zinc oxide support, while we did not observe any peak indicative of a palladium *fcc* phase (Figure S6).

Figure S7a–c shows TEM images of fresh 2Pd‐ZnO‐np catalyst. A fast Fourier transform (FFT) of TEM image shown in Figure S7c revealed several spots corresponding to different crystal lattice vectors of zinc oxide and palladium oxide. By applying circular masks at a given lattice spot in the FFT of the 2Pd‐ZnO‐np sample (Figure S7d), and then calculating the inverse FFT (IFFT), we have determined the locations of the corresponding ZnO and PdO phases. Figure S7e shows superposition of original micrograph of 2Pd‐ZnO‐np sample with IFFT of Figure S7d, where circular masks were applied at lattice spacing of 1/2.60 Å^−1^, corresponding to the (002) planes of zinc oxide (red), and at a lattice spacing of 1/2.63 Å^−1^, which corresponds to the (101) planes of palladium oxide (blue). Although these d‐values are rather similar, the darker contrast of the smaller particles indicate the presence of the PdO phase there (mass contrast).

TEM images of spent 2Pd‐ZnO‐np catalyst and corresponding FFT and IFFT (Figure S8) reveal the formation of PdZn alloy phase after catalytic carbon dioxide hydrogenation. An FFT of the area represented on Figure S8a also shows two spots at 1/2.60 Å^−1^ and 1/1.63 Å^−1^ which belong to the (002) and (110) planes of wurtzite zinc oxide phase. Further reflections, at 1/2.05 Å^−1^ and 1/2.20 Å^−1^ correspond, to (200) and (111) planes of the palladium–zinc alloy and match those of the (200) and (111) d‐spacing obtained from XRD and TEM examinations by Malik et al.^[31]^ The reconstructed IFFT images also show the localization of the zinc oxide phase (Figure S8c), where circular masks were applied at 1/2.60 Å^−1^ and 1/1.63 Å^−1^, as well as that of PdZn alloy nanoparticle (Figure S8d), where circular mask was applied at 1/2.05 Å^−1^ corresponding to (200) planes of palladium–zinc alloy.

To summarize the results of these operando experiments, catalytic carbon dioxide hydrogenation occurs via a formate intermediate route. FTIR spectra suggest that the active formate species are bound to zinc (characteristic bands at 1596 cm^−1^ and 1376 cm^−1^). XAS revealed the absence of palladium–zinc de‐alloying or any indication of the surface oxidation of palladium, which might be expected due to possible palladium‐formate intermediates, during the catalytic cycle. Instead, transient experiments show that the palladium–zinc phase is very stable under different gas atmospheres (hydrogen, CO_2_/H_2_ mixture and carbon dioxide) and it may well be only responsible for hydrogen activation, while carbon dioxide activation, and its subsequent hydrogenation, occur on the zinc oxide similar to the Cu/ZnO‐based system.[^25^, ^26^] However, at this point, it is not clear, whether the palladium–zinc alloy alone governs the activity and selectivity of the catalyst, or the interface between the alloy phase and bulk zinc oxide represents the active site for the selective transformation of carbon dioxide to methanol.

To investigate the role of palladium–zinc alloy in this system, we have designed the following experiment to distinguish the activity of palladium–zinc alloy from that of the PdZn/ZnO interface in this reaction. From operando XAS and XRD experiments, we have found that, under carbon dioxide hydrogenation conditions, palladium is present in the form of a palladium–zinc alloy. Therefore, we supported a heterobimetallic Pd^II^Zn^II^ acetate bridge complex on silica and alumina and reduced it to obtain palladium–zinc alloy supported catalysts,[^30^, ^32^, ^33^] PdZn/SiO_2_ and PdZn/Al_2_O_3_, which do not contain any zinc oxide, and therefore cannot present a PdZn/ZnO interface. Alternatively, a palladium–zinc catalyst with excess of ZnO (i.e. PdZn/ZnO/SiO_2_) was synthesized by a double impregnation of silica, firstly with the Pd^II^Zn^II^ acetate complex, and secondly with zinc nitrate. This sample was then calcined in air and then reduced in hydrogen, such that the role of zinc oxide and of the PdZn/ZnO interface on the catalysis could be determined. Both samples (i.e. PdZn/ZnO/SiO_2_ and PdZn/ZnO/SiO_2_) possess similar particle size distributions, comparable to that of 2Pd‐ZnO‐i (Figures S11,S12).

**Figure 3:**
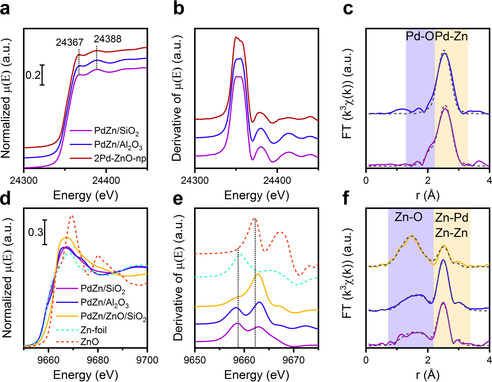
Pd K‐edge XANES (a), the first derivatives of corresponding XANES spectra (b) as well as the corresponding Fourier transforms (c) of the k^3^‐weighted data of PdZn/SiO_2_, PdZn/Al_2_O_3_ and 2Pd‐ZnO‐np catalysts collected in situ after reduction in hydrogen at 260 °C and 15 bar pressure. Zn K‐edge XANES (d), the first derivatives of corresponding XANES spectra (e) as well as the corresponding Fourier transforms (f) of the k^3^‐weighted data of PdZn/SiO_2_, PdZn/Al_2_O_3_, and PdZn/ZnO/SiO_2_. The latter all measured in situ post reaction after catalytic carbon dioxide hydrogenation (260 °C, 30 bar).

Figure 3 a,b shows Pd K‐edge XANES, and the first derivatives thereof, of PdZn/SiO_2_ and PdZn/Al_2_O_3_ after calcination and reduction, as well as the spectrum of the 2Pd‐ZnO‐np catalyst discussed earlier. Pd K‐edge XANES spectra of samples prepared via the Pd^II^Zn^II^ acetate route resemble those of both 2Pd‐ZnO‐np and a palladium foil standard. According to EXAFS analysis (Figure 3 c and Table S4), both of these systems are characterized by a Pd‐Zn scattering shell at ca. 2.57–2.6 Å. As such, the Pd K‐edge XANES and EXAFS in both of these cases confirms the formation of a palladium–zinc alloy (see Figure S13 and corresponding discussion in the Supporting Information).

From Zn K‐edge XANES (Figure 3 d), the most significant variation observed between these three systems (PdZn/SiO_2_, PdZn/Al_2_O_3_ and PdZn/ZnO/SiO_2_) lies in the magnitude of the low binding energy feature at ca. 9658 eV. This feature we may associate with the formation of either, reduced zinc (Zn^0^), and therefore the presence of palladium–zinc alloys, and/or the presence of zinc oxygen vacancies.^[34]^ The double peak structure of the derivative spectra (Figure 3 e) could be indicative of the presence of both Zn^0^ and Zn^II^ containing phases in each of the catalyst systems. We might further intuit, from the relative magnitudes of the pre‐edge feature (Figure 3 d), that the proportions of the two zinc states are markedly different across the samples, with significantly higher levels of reduced zinc being indicated for PdZn/SiO_2_ and PdZn/Al_2_O_3_ compared to PdZn/ZnO/SiO_2_.

For PdZn/SiO_2_, PdZn/Al_2_O_3_ the phase‐corrected FTs (Figure 3 f) are dominated by the scattering shell at ca 2.6 Å that we may again associate (see also Table S5) with the significant presence of the palladium–zinc alloy phase. In these two cases, this is accompanied by a very broad feature in the FT, indicative of low Z (modelled as oxygen) coordination to some fraction of the zinc atoms. Given that the XANES from these two cases (Figure 3 d) points to a predominance of zinc being present in a reduced form, this rather significant level of coordination might suggest a segregation of the zinc toward the surface of the alloy nanoparticles particles where they may be in contact with either reactant species or the underlying support. Different from both of these cases is the PdZn/ZnO/SiO_2_. Consistent with the reduced intensity of the low binding energy pre‐edge feature observed in XANES (Figure 3 d), the EXAFS, in this case, is dominated by a broad low Z (O) scattering shell (Figure 3 f and Figure S14). We also find the shell at ca 2.6 Å, indicative of the presence of the palladium–zinc alloy, but at a much reduced level in terms of its contribution to the overall EXAFS envelope. Together with the XANES (Figure 3 d) and TEM (Figures S11,S12), this sample is therefore comprised of both palladium–zinc alloy and zinc oxide phases, with a much heavier weighting to the latter than the former. Overall, we found that palladium–zinc alloy phase in these samples is stable during carbon dioxide hydrogenation, and similarly to 2Pd‐ZnO‐np sample, does not undergo oxidation or de‐alloying during methanol synthesis.

Table 2 shows results of catalytic carbon dioxide hydrogenation for the PdZn/SiO_2_ and PdZn/Al_2_O_3_ samples. Despite significant carbon dioxide conversion (2.6–4.4 %), both materials produce carbon monoxide as the main product, and methanol selectivity for PdZn/SiO_2_ and PdZn/Al_2_O_3_ catalysts is correspondingly low, at 11 and 6 % respectively. Thus, methanol productivity for both materials is below 30 g_MeOH_ kg_cat_^−1^ hour^−1^, which is significantly lower than attained in the 2Pd‐ZnO‐np case (vide supra). These results permit a fundamental conclusion to be drawn: that the presence of the palladium–zinc alloy phase of itself does not guarantee that the selective hydrogenation of carbon dioxide to methanol will result. PdZn/SiO_2_ and PdZn/Al_2_O_3_ exhibit significant activity in the RWGS reaction, which produces carbon monoxide and water.

**Table 2:** Result of catalytic carbon dioxide hydrogenation over different catalysts.^[a]^

Catalyst	CO_2_ conv. [%]	Methanol productivity [g_MeOH_ kg_cat_^−1^ h^−1^]	Methanol selectivity [%]
PdZn/SiO_2_	2.6	30	11.2
PdZn/Al_2_O_3_	4.4	27	6.0
PdZn/ZnO/SiO_2_	3.6	184	49.8
PdZn/ZnO/SiO_2_^[b]^	3.3	443	65.3

[a] Unless otherwise indicated, catalytic experiments were performed at 260 °C and 30 bar, catalyst mass: 100 mg, CO_2_:H_2_ ratio equals 1:3 and flowrate: 50 mL min^−1^. [b] 2Pd‐ZnO‐np and PdZn/ZnO/SiO_2_ catalysts were also compared at 260 °C and 50 bar total pressure, catalyst mass: 50 mg, CO_2_:H_2_ ratio equals 1:3 and flowrate: 50 mL min^−1^.

The addition of zinc oxide leads to a much improved selectivity to methanol (Table 2) and PdZn/ZnO/SiO_2_ produces six times more methanol per catalyst mass than the PdZn/Al_2_O_3_ or PdZn/SiO_2_. Though similar, in terms of carbon dioxide conversion (3.6 % at 30 bar total pressure), the PdZn/ZnO/SiO_2_ sample, produces 184 g_MeOH_ kg_cat_^−1^ hour^−1^ as a result of the greatly enhanced selectivity to methanol (49.8 %). Furthermore, at 50 bar pressure, the performance level achieved in the PdZn/ZnO/SiO_2_ case, in terms of activity and selectivity toward the production of methanol, is very similar, and even slightly better, to that achieved using a bulk zinc oxide support (the 2Pd‐ZnO‐np sample). Thus, considering the results of the operando study of the 2Pd‐ZnO‐np catalyst, we can conclude that palladium–zinc alloy itself does not provide the active sites required to produce methanol from the direct hydrogenation of carbon dioxide. Instead, we have shown that zinc oxide phase is responsible for carbon dioxide activation, while the palladium–zinc alloy in this bifunctional catalyst activates hydrogen and enables the further hydrogenation of the formate on zinc to methanol.[^25^, ^28^] Our data therefore points to selective catalytic activity taking place predominantly on the zinc oxide and likely at the interface with the palladium–zinc alloy.

## Conclusion

In contrast to copper‐zinc alloys, we have shown through the application of operando XAS, that nanoparticulate palladium–zinc is stable and does not undergo oxidative disruption under the conditions required for the direct hydrogenation of carbon dioxide to methanol. More significantly, however, through comparing the behavior of catalyst systems specifically designed to yield nanoparticulate palladium–zinc in the absence or presence of a zinc oxide phase, we have shown that the palladium–zinc alloy phase by itself is not particularly good at selectively hydrogenating carbon dioxide to methanol. Instead, in the absence of a co‐existing zinc oxide phase, the palladium–zinc alloy primarily yields carbon monoxide. When zinc oxide and the palladium–zinc alloy nanoparticles are both present, the system becomes a far more active and selective catalyst. At the highest pressures used in this work, a catalyst based on zinc oxide and palladium–zinc alloy supported on silica matches the methanol synthesis performance of typical Pd/ZnO catalysts.

These data show that carbon dioxide hydrogenation to methanol requires a multifunctional catalyst, with zinc oxide activating carbon dioxide, and the palladium–zinc alloy splitting hydrogen. In this system, as with the archetypal Cu/ZnO/Al_2_O_3_ case, selective methanol synthesis is therefore the result of a synergy that exists between an oxidized zinc phase, upon which active formates may be hosted, and a metallic phase whose primary function is to dissociate hydrogen. This hydrogen may then spillover to affect the selective hydrogenation of the formates and result in the desired synthesis of methanol, rather than the undesired formation carbon monoxide.

## Conflict of interest

The authors declare no conflict of interest.

## Supporting information

As a service to our authors and readers, this journal provides supporting information supplied by the authors. Such materials are peer reviewed and may be re‐organized for online delivery, but are not copy‐edited or typeset. Technical support issues arising from supporting information (other than missing files) should be addressed to the authors.

Supplementary
