# Supplementary material for: Mechanistic Study of Carbon Dioxide Hydrogenation over Pd/ZnO‐Based Catalysts: The Role of Palladium–Zinc Alloy in Selective Methanol Synthesis
Source: Angew Chem Int Ed Engl. 2021 Jun 24;60(31):17053–9. doi: 10.1002/anie.202103087 (PMC8361960; doi:10.1002/anie.202103087)
Supplement: Supplementary file 1 — Supplementary [file ANIE-60-17053-s001.pdf]

Angewandte Chemie
Eine Zeitschrift der Gesellschaft Deutscher Chemiker

Supporting Information

Mechanistic Study of Carbon Dioxide Hydrogenation over Pd/ZnO-Based Catalysts: The Role of Palladium–Zinc Alloy in Selective Methanol Synthesis

Maxim Zabilskiy, Vitaly L. Sushkevich, Mark A. Newton, Frank Krumeich, Maarten Nachtegaal, and Jeroen A. van Bokhoven**

anie_202103087_sm_miscellaneous_information.pdf

Materials and Methods

Catalyst preparation

Synthesis of palladium nanoparticles

Monodispersed palladium nanoparticles were synthesized according to the following protocol using Schlenk techniques. 121.8 mg of palladium acetylacetonate (99 %, Sigma-Aldrich), 20 ml of octadecene (technical grade, Sigma-Aldrich), and 1.32 ml of oleylamine (technical grade, Sigma-Aldrich), were mixed in a three-neck flask. The mixture was evacuated at room temperature for 30 min under magnetic stirring. After that, the reaction flask was flushed with nitrogen and 0.446 ml of trioctylphosphine (97 %, Sigma-Aldrich) was added under stirring. The mixture was heated to 70 °C and the transparent solution was left under vacuum for another 30 min. The reaction flask was then flushed with nitrogen and heated to 250 °C (heating ramp ~40 °C min^{-1}) using a pre-heated sandbath. The solution became black as a result of the formation of palladium nanoparticles and, after 15 min of reaction, the mixture was quickly cooled down to RT using a compressed air stream. Palladium nanoparticles were precipitated by addition of an ethanol/acetone mixture (1:1), and then separated by centrifugation (10000 rpm, 10 min). Purification was performed by three rounds of re-dissolution of nanoparticles in hexane (20 mL) followed by precipitation with an ethanol/acetone mixture and centrifugation. Finally, the palladium nanoparticles were dissolved in 20 ml of hexane, producing a deep black solution, and stored at RT.

Synthesis of ZnO support

5.8 g of Pluronic P123 (99%, Sigma-Aldrich) were dissolved in 170 mL of distilled water under vigorous stirring at room temperature. 2.2 g of zinc acetate dihydrate (99 %, Sigma-Aldrich) and 3 g of urea (99 %, Alfa Aesar) were added to this solution, while stirring for another 30 min at ambient temperature. Finally, the solution was poured into a 150 mL Teflon lined autoclave, and hydrothermal synthesis was performed at 90 °C. After 24 hours the autoclave was cooled down to room temperature and the white precipitate thoroughly washed with distillated water, dried overnight at 110 °C, and finally calcined in air at 350 °C (heating ramp 2 °C/min) for 4 hours. The resulting zinc oxide powder was then stored and used for preparation of Pd/ZnO catalysts.

Synthesis of 2Pd-ZnO-np

300 mg of the zinc oxide support prepared during the previous step was impregnated with 2 ml of a solution containing palladium nanoparticles in hexane. This sample was dried overnight at room temperature and then calcined in air at 350 °C (heating ramp 2 °C/min) for 4 hours. As synthesized, this material contained 2.04 wt. % of palladium (according to AAS analysis) and it henceforth denoted as 2Pd-ZnO-np.

Synthesis of 2Pd-ZnO-i

The 2Pd-ZnO-i sample was prepared by incipient wetness impregnation of the same zinc oxide support used in the previous step using an aqueous palladium nitrate solution. 300 mg of zinc oxide was impregnated with 2 ml of solution containing 15.3 mg of palladium nitrate dehydrate (99.9 %, ABCR). The sample was again dried overnight at room temperature, prior to being calcined in air at 350 °C (heating ramp 2 °C/min) for 4 hours. This material, denoted as 2Pd-ZnO-i, contained 1.95 wt. % of palladium (accordingly to AAS analysis).

Synthesis of heterobimetallic $Pd^{II}Zn^{II}$ acetate bridge complex

The heterobimetallic lantern complex, $PdZn(CH_3COO)_4{\cdot}H_2O$, was synthesized according to previous reports.[32,35] A slurry containing 448 mg of palladium acetate (98 %, ABCR) and 440 mg of zinc acetate dehydrate (99 %, Sigma-Aldrich) in 20 ml of acetic acid (99 %, Sigma-Aldrich) was stirred at 90 °C until a complete dissolution of inorganic salts was attained. The resulting clear solution was then cooled to room temperature and acetic acid was removed using a rotary evaporator. In order to remove any remaining residue of acetic acid, the powder was then dissolved in tetrahydrofuran and the solvent removed, again using rotary evaporation. Finally, the $Pd^{II}Zn^{II}$ acetate bridge complex was dissolved in 20 ml of a tetrahydrofuran/methanol mixture (3:1), and immediately used for preparation of palladium-zinc based catalysts through impregnation of the $Pd^{II}Zn^{II}$ acetate bridge complex on silica and alumina supports.

Synthesis of $PdZn/SiO_2$

1 g of silica (Aldrich, LOT: MKBS9858V) was impregnated with 5.1 ml solution of the $PdZn(CH_3COO)_4$ complex prepared during the previous step. The resulting sample was dried at room temperature for 1 hour, then at 100 °C for 1 hour, before finally being calcined in air at 350 °C (heating ramp 2 °C/min) for 4 hours. This material, denoted as $PdZn/SiO_2$ contained 4.15 wt. % of palladium and 2.55 wt. % of zinc (accordingly to AAS analysis).

Synthesis of $PdZn/Al_2O_3$

1 g of γ-alumina was prepared by calcination of Boehmite (see reference [37] for details) and then impregnated with 5.1 ml solution of $PdZn(CH_3COO)_4$. This sample was also dried at room temperature for 1 hour, then at 100 °C for 1 hour prior to calcination in air at 350 °C (heating ramp 2 °C/min) for 4 hours. The resulting sample, denoted as $PdZn/Al_2O_3$, contained 4.08 wt. % of palladium and 2.46 wt. % of zinc (accordingly to AAS analysis).

Synthesis of $PdZn/ZnO/SiO_2$ sample

300 mg of $PdZn/SiO_2$ sample was reduced in hydrogen at 260 °C for 1 hour and then cooled to room temperature. The sample was then impregnated with 2.5 ml aqueous solution that contained 115 mg of zinc nitrate hexahydrate (98 %, Sigma-Aldrich). The sample was, as before, dried overnight at room temperature, before being calcined in air at 350 °C (heating ramp 2 °C/min) for 4 hours. This material, henceforth denoted as $PdZn/ZnO/Al_2O_3$ contained 3.74 wt. % of palladium and 9.91 wt. % of zinc (accordingly to AAS analysis).

2.2. Catalyst characterization

Operando XAS and XRD

Combined *operando* X-ray diffraction and X-ray absorption spectroscopy studies were performed at the Swiss-Norwegian BM31 beamline at the European Synchrotron Radiation Facility (ESRF, Grenoble, France).[38] About 10 mg of catalyst (fraction 50-100 μm) was positioned inside the 2 mm, (wall thickness 0.05 mm) quartz capillary reactor, and fixed between two quartz wool plugs. A modified version of the plug-flow reactor system developed by Chupas and co-workers for total X-ray scattering measurements was used during these experiments.[39] Temperature was controlled by using a K- type thermocouple inserted into the sample bed. Gas flows (2-10 ml min^{-1}) were controlled by mass flow controllers (Bronkhorst). The total pressure was controlled by a Bronkhorst EL-Press digital back-pressure regulator. All gases (hydrogen, carbon dioxide, argon, helium) used were of grade 6.0 purity. Transient switches were performed by using a remote controlled 6-port 2-position valve (VICI, Valco Instruments).

Combined Pd K-edge and Zn K-edge XAS and XRD, measurements were made in a transmission geometry using two, rapidly interchangeable monochromators; a high-resolution, powder diffraction-dedicated, channel-cut monochromator (CCM), and a second, double crystal, monochromator (DCM) for XAFS. A metal-oxide semiconductor (CMOS) detector, with an active area of 290.8 x 229.8 mm was used to obtain X-ray powder diffraction,[40] whilst ion chambers were used for measurement of the XAS. A XAS spectrum of palladium foil, collected in parallel

with the XAS from the sample, was used for internal energy calibration. Transient responses of the catalyst, upon switching the reaction mixture, were monitored by means of an Omnistar GSD 300 O2 (Pfeiffer Vacuum) mass spectrometer.

Further *in situ* XAS measurements were then also made, at the Zn K-edge and Pd K-edges of $PdZn/SiO_2$, PdZn/ Al_2O_3 and PdZn/ZnO/ SiO_2 catalysts at the SuperXAS beamline, Swiss Light Source of the Paul Scherrer Institute (Villigen, Switzerland). After catalytic carbon dioxide hydrogenation, or after pre-treatment in hydrogen (see Figure captions for details), the aforementioned samples were transferred to a glovebox without exposure to air. Samples were then packed into either 2 mm (Pd K-edge) or 1 mm (Zn K-edge) internal diameter quartz capillaries and sealed using epoxy-glue. XAS data were collected in transmission using fast, gridded ion chambers, and a quick scanning channel-cut Si (111) monochromator (oscillation frequency of 1 Hz).[41] Either zinc or palladium foil standards were collected simultaneously for energy calibration. Initial analysis and energy calibration were performed using ProXAS v.2.34 software.[42] Zn K- and Pd K-edge XANES and EXAFS data were background subtracted and normalized using either, PAXAS,[43] Athena,[44] or Prestopronto.[45] Principal component analysis (PCA) was made using the ITFA software developed by Rossberg et al.[46] Fitting of the EXAFS was made using EXCURV (v. 9.3).[31]

Operando steady-state $^{12}CO_2/^{13}CO_2$ *isotope transient experiment coupled with infrared spectroscopy*

Time-resolved isotope transient experiments coupled with FTIR were performed using a standard sandwich flow IR cell reactor configuration.[47] Before the measurements, the sample was diluted with pure silica (1:3 by weight), pressed in a self-supporting disk, and activated in helium for 2 hours at 260 ° C. (heating rate 10 °C./min). The sample was subsequently reduced in hydrogen (25 ml/min) for 1 hour at 260 °C under ambient pressure. The gas flow was then switched from hydrogen to a CO_2/H_2/Ar mixture (24 vol. %, 72 vol. % and 4 vol. % correspondingly, Messer). Finally, the total pressure was increased to 15 bar (regulated by a Bronkhorst back-pressure regulator, EL-Press series). After reaching steady state carbon dioxide conversion (ca. 1 h on stream), the isotope switch was carried out using a two-position valve (VICI), from unlabeled $^{12}CO_2/H_2$ (Messer; 99.5%) to a $^{13}CO_2/H_2$ labeled mixture (Cambridge Isotopes Laboratories, Inc.; 99% ^{13}C). An inert tracer (Argon, 4 vol. % of the total flow rate) was used to correct for the gas-phase hold-up in the reactor. Isotope labeling experiments were performed at a constant total gas flow of 25 ml/min. The $^{12}C/^{13}C$ switch was achieved without perturbing the steady-state of the reaction by maintaining the reaction temperature at 260 °C, the total system pressure at 15 bar, and

at carbon dioxide conversion level of ≈2%. On-line MS analysis was performed using a quadrupole mass analyzer (Omnistar GSD 300 O2). The surface species on the catalyst were followed using IR spectroscopy (Thermo Nicolet iS50 equipped with MCT detector). The spectral resolution was 4 cm^{-1}, and spectra were acquired every 6 secs (by averaging of 4 scans per spectrum).

In the MS, the ion current for m/z = 40 (Ar tracer), 44, 45, 28, 29, 31 and 33 were continuously monitored to determine the isotope content of the original gas sample. Transient responses were normalized by the difference between the initial and final ion signals. The argon decay curve was used to determine the gas-phase holdup of the reactor system, since we assumed that the inert gas did not adsorb on the surface of the catalyst.

Microscopy

For the (scanning) transmission electron microscopy ((S)TEM) investigations, the material was dispersed in ethanol and a few drops of the suspension were deposited onto a perforated carbon foil supported on a Cu TEM grid. After evaporation of the ethanol, the grid was mounted on the single tilt holder of the microscope. TEM and STEM combined with energy-dispersive X-ray spectroscopy (EDXS) was performed on a Talos F200X microscope (ThermoFisher) with a high brightness field emission gun operated at an acceleration potential of 200 kV. The EDX system of this microscope consists of 4 silicon drift detectors (SDD), which enables one to record EDXS maps with good a signal-to-noise ratio in a relatively short collection time (10-20 min).

2.3. Catalytic experiments

Catalytic methanol synthesis over palladium-zinc based catalysts was also investigated using a fixed-bed stainless-steel reactor. In a typical experiment, 25 or 50 mg of the catalyst (fraction 50-100 μm), diluted (1:3 by mass) with silicon carbide (Sigma-Aldrich), was loaded between two quartz wool beads and positioned inside a stainless-steel tube reactor (6 mm outer diameter and 4 mm inner diameter). The reactor was mounted inside a single-zone furnace (Carbolite). The temperature was controlled with a Eurotherm 3508 controller using a K-type thermocouple positioned inside the catalyst-bed. The catalyst was activated firstly, under ambient pressure in a flow of argon (5.0 quality, 50 mL/min) at 260 °C (heating rate 5 °C/min) for 2 h, and secondly, by a reduction in hydrogen (50 mL/min) at 260 °C and ambient pressure for 1 hour. Finally, the total pressure was increased to 30 or 50 bar using a back-pressure regulator (Bronkhorst, EL-PRESS). Carbon dioxide hydrogenation catalysis was initiated by switching the feedstock from pure hydrogen to a feed gas mixture containing 24 vol. % of carbon dioxide, 72 vol. % of hydrogen, and 4 vol. % of argon (all gases used were of 5.0 quality, Messer) at 260 °C (see details in Table 1). After 24 hours on stream, the catalytic properties of the material were further investigated under

different temperatures (250, 240 and 220 °C). After any temperature changes, the catalyst was equilibrated for ca. 1 hour until a steady-state methanol yield was achieved. Catalytic data were then acquired for a further 1 hour. Analysis of outlet gases was performed using a 3000 Micro GC gas analyzer (Inficon) equipped with a 10 m Molsieve and an 8 m PlotU columns and TCD detectors.

Table S1. Comparison of catalytic carbon dioxide hydrogenation over Pd/ZnO based catalysts.

Catalyst	CO_2:H_2 ratio	Temperature (°C)	Pressure (bar)	Carbon dioxide conversion (%)	Methanol selectivity (%)	Methanol space time yield ($g_{MeOH}\cdot kg_{cat}^{-1}\cdot hour^{-1}$)	Reference
5 wt. % Pd/ZnO	1:3	250	39	13.7	57	385	[48]
10 wt. % Pd/ZnO	1:9	190	1	N/A	65.1	2.5	[12]
4.9 wt. % Pd/ZnO	1:3	260	30	8.4	62.3	N/A	[49]
16 wt. % $Pd_{0.1}Zn_1$/CNTs	1:3	250	30	7.3	86.3	37.1	[13]
10 wt. % $Pd_{0.1}Zn_1$/CNTs	1:3	270	50	19.91	31.4	307	[14]
5 wt. % Pd/ZnO	1:3	180	30	2.5	72.2	21.4	[7]
5 wt. % Pd/ZnO/Al_2O_3				2.9	79.4	27.2	
5 wt. % Pd/ZnO	1:3	250	20	10.7	60	77.4	[5]
10.9 wt. % Pd/ZnO	1:9	250	1	2.45	15.79	4.1	[50]
5 wt. % Pd/ZnO	1:3	250	20	8.2	32	34.2	[24]
5 wt. % Pd_1Zn_5/TiO_2				10.1	40	55.4	
2Pd-ZnO-np (2 wt. % Pd/ZnO)	1:3	260	50	2.8	66.5	384	This study
PdZn/ZnO/SiO_2				3.3	65.3	443	

Table S2. Comparison of catalytic carbon dioxide hydrogenation over Pd/ZnO based catalysts.

Material	Frequency (cm^{-1})	Vibration mode	Band assignment	Reference
Pd/ZnO-CdSe	1595<br>1395	δ_{as}(HCOO)<br>δ_s(HCOO)	formate	[51]
Pd/Ga_2O_3	1660<br>1600<br>1580	δ_{as}(HCOO)<br>δ_{as}(HCOO)<br>δ_{as}(HCOO)	monodentate formate<br>bidentate formate<br>bridge formate	[52]
Pd/ZrO_2	1580-1590<br>1370	δ_{as}(HCOO)<br>δ_s(HCOO)	surface formate	[53]
Pd/β-Ga_2O_3	1640<br>1298<br>1580<br>1372	δ_{as}(HCOO)<br>δ_s(HCOO)<br>δ_{as}(HCOO)<br>δ_s(HCOO)	monodentate formate<br>monodentate formate<br>bidentate formate<br>bidentate formate	[54]
Cu/ZnO/Al_2O_3	1596<br>1372	δ_{as}(HCOO)<br>δ_s(HCOO)	surface zinc formate	[27]

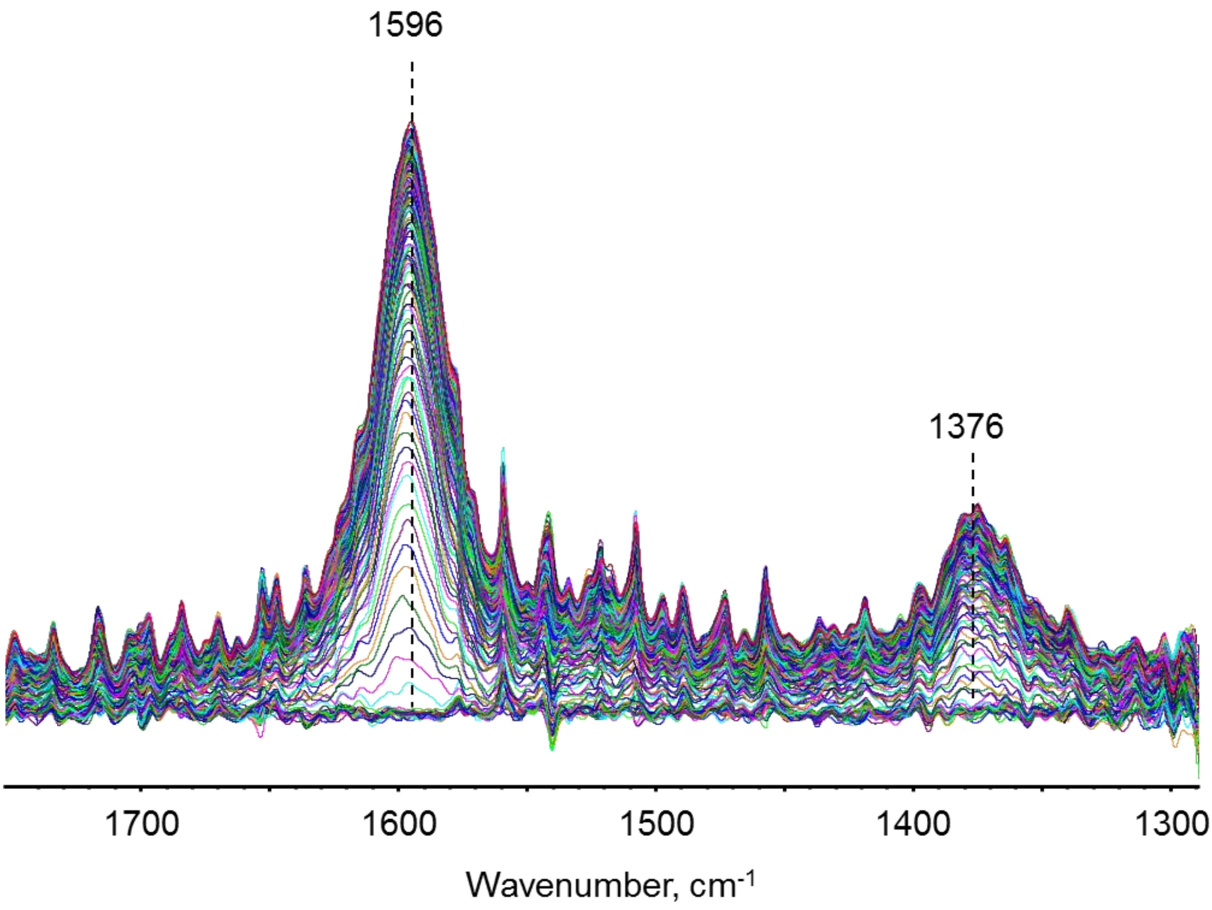

Figure S1. Time-resolved FTIR difference spectra acquired during the switch from H_2 to $^{12}CO_2/H_2$ mixture at 15 bar and 260 °C after reduction for 30 min. The time resolution of IR spectra is 6 seconds, the measurement was performed in transmission mode. The bands at 1596 and 1376 cm^{-1} correspond to the asymmetric and symmetric vibrations in isotopically unlabeled formate species, respectively. No other pronounced bands were detected.

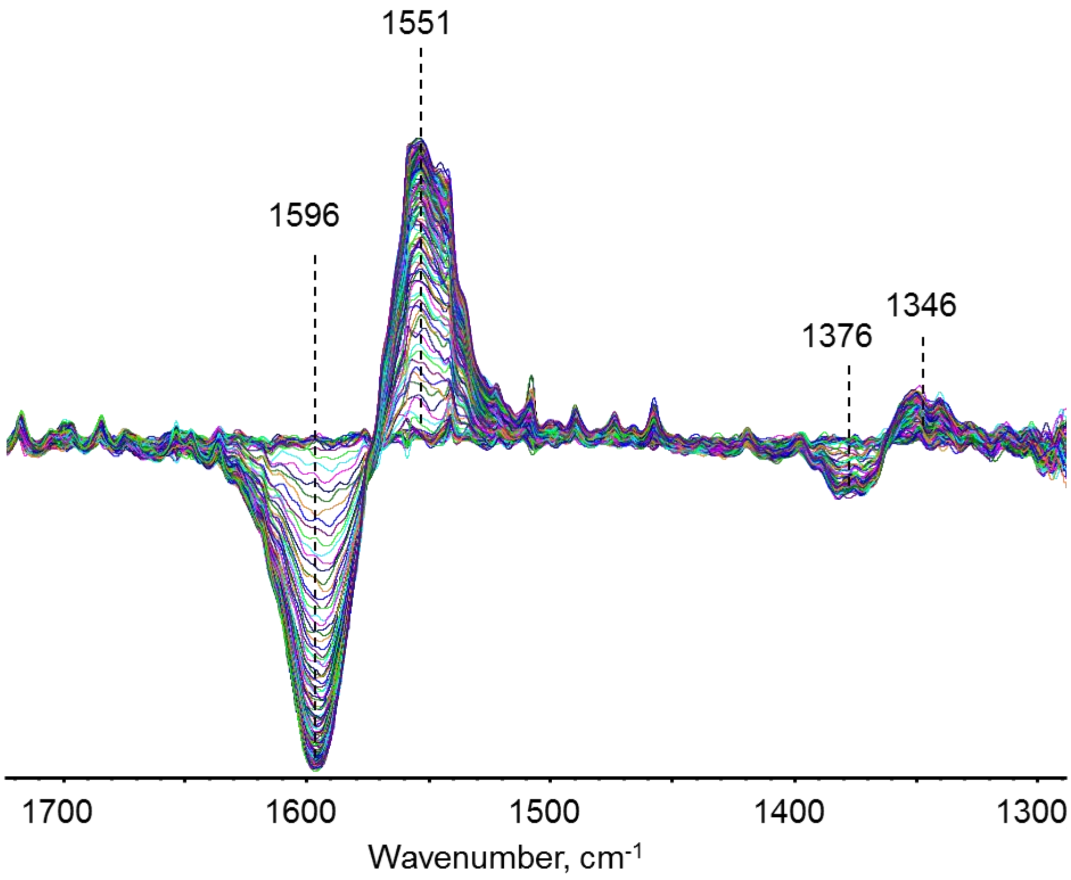

Figure S2. Time-resolved FTIR difference spectra acquired during the switch from $^{12}CO_2/H_2$ mixture to $^{13}CO_2/H_2$ mixture at 15 bar and 260 °C after system equilibration for 1 h. The time resolution of IR spectra is 6 seconds, the measurement was performed in transmission mode. The bands at 1596 and 1376 cm^{-1} correspond to the asymmetric and symmetric vibrations in isotopically unlabeled formate species, respectively. Switch to $^{13}CO_2$-containing reaction feed leads to the compete depletion of ^{12}C-formate with simultaneous formation of ^{13}C-formates, characterized by the bands at 1551 and 1346 cm^{-1}.

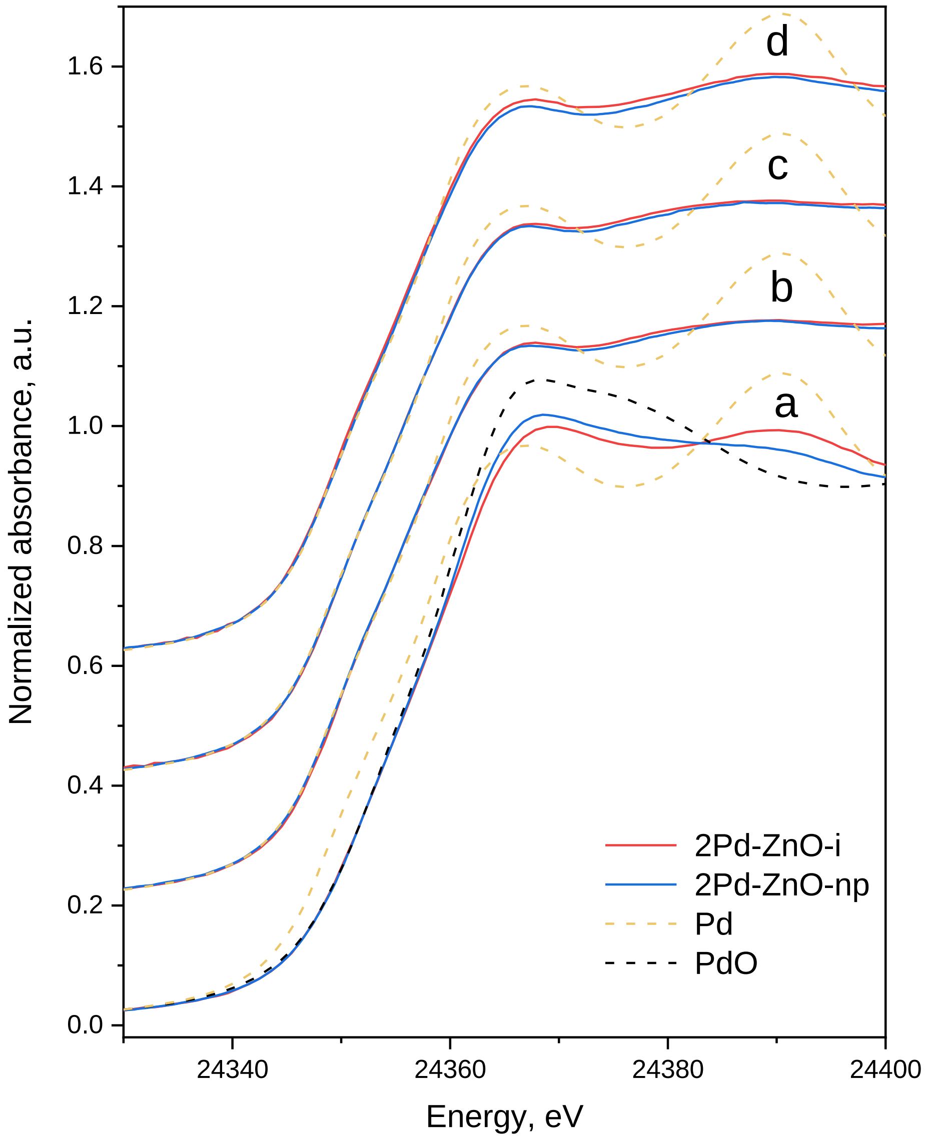

Figure S3. Pd K edge XANES spectra collected during activation and catalytic carbon dioxide hydrogenation at 260 °C and 15 bar pressure over 2Pd-ZnO-i (red) and 2Pd-ZnO-np (blue) catalysts: in helium (a), after reduction in hydrogen (b), during catalytic carbon dioxide hydrogenation (c) and after switch to pure carbon dioxide (d); as well as XANES spectra of reference standards: palladium foil (gold) and palladium oxide (black).

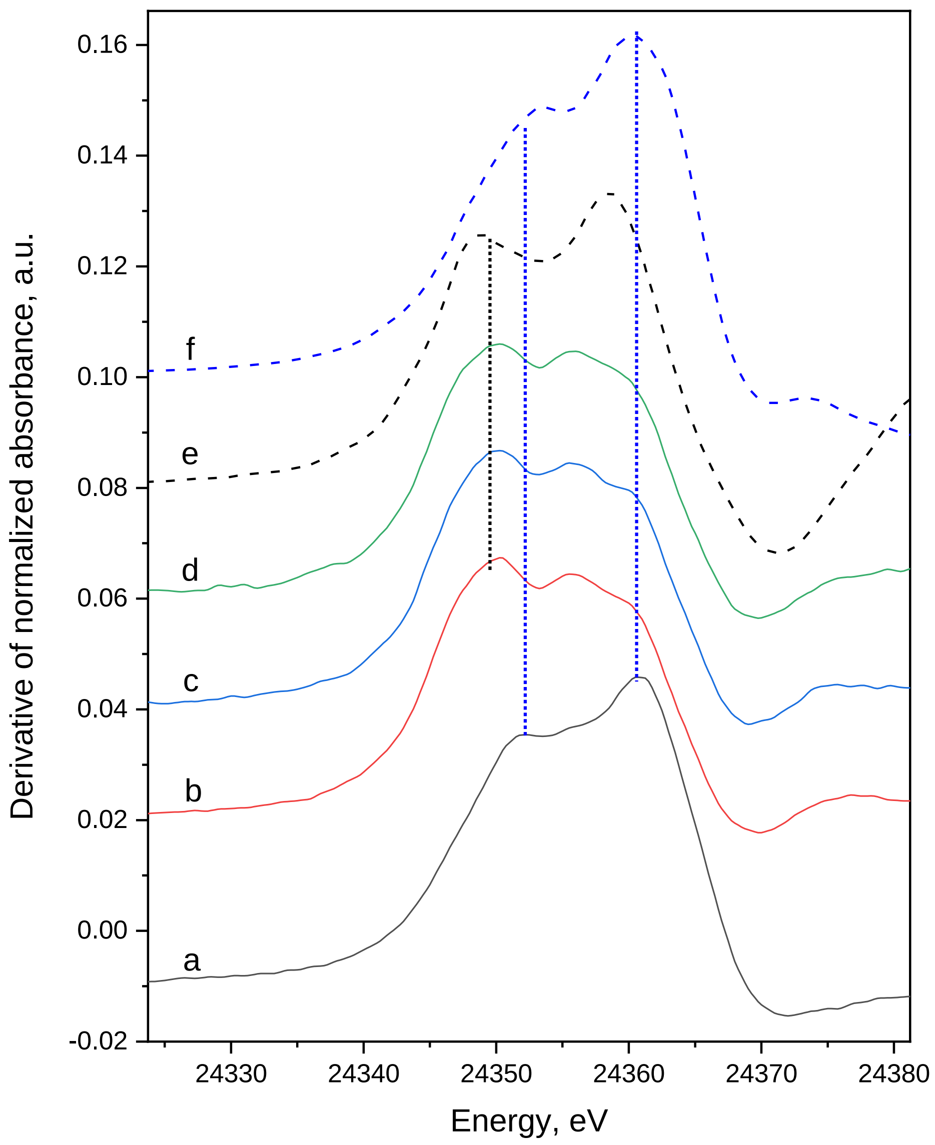

Figure S4. First derivative of XANES spectra collected during activation and catalytic carbon dioxide hydrogenation at 260 °C and 15 bar pressure over 2Pd-ZnO-np catalyst: in helium (a), after reduction in hydrogen (b), during catalytic carbon dioxide hydrogenation (c) and after switch to pure carbon dioxide (d); as well as first derivative of reference standards: palladium foil (e) and palladium oxide (f).

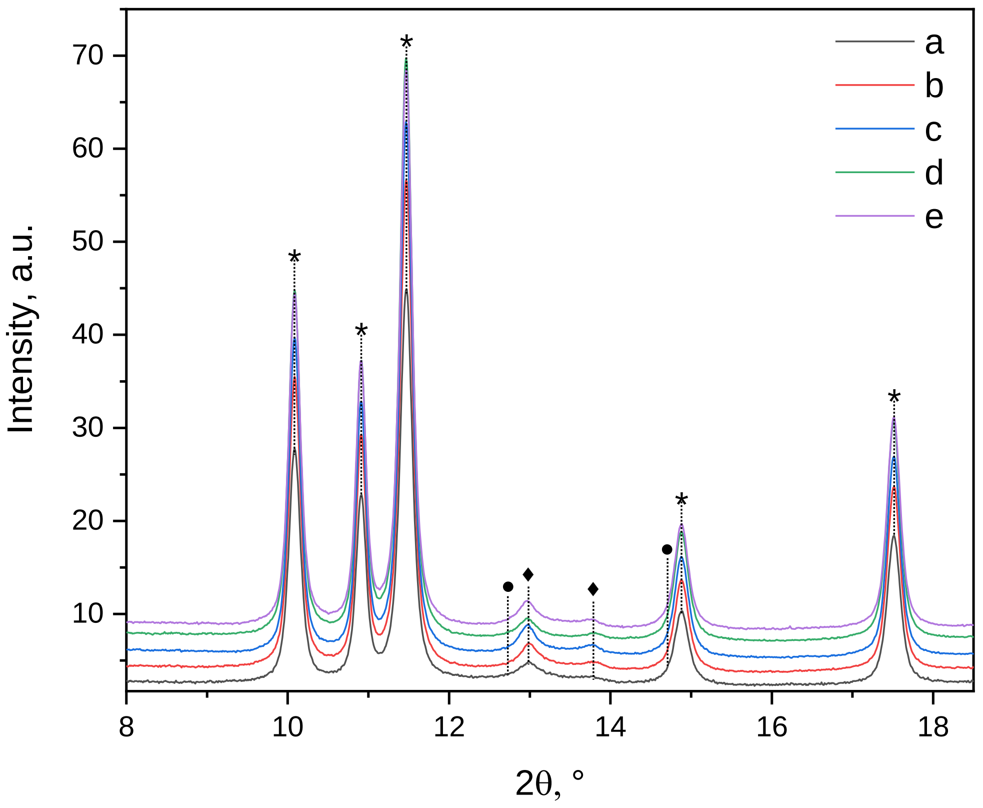

Figure S5. XRD patterns of 2Pd-ZnO-i catalyst collected during combined operando XAS-XRD study at 260 °C and 15 bar (λ= 0.4975 A) under different gas atmospheres: in hydrogen (a); after transient switch to CO_2/H_2 mixture (b); in hydrogen after transient switch from CO_2/H_2 mixture; (c) in carbon dioxide after transient switch from CO_2/H_2 mixture (d); as well as in CO_2/H_2 mixture after transient switch from carbon dioxide (e). Diffraction peaks marked with asterisk (*) correspond to zinc oxide wurtzite phase. Diffraction peaks of PdZn alloy are labeled with diamond symbol (♦). Positions of diffraction peaks where pure palladium phase can be observed are marked with circle (●).

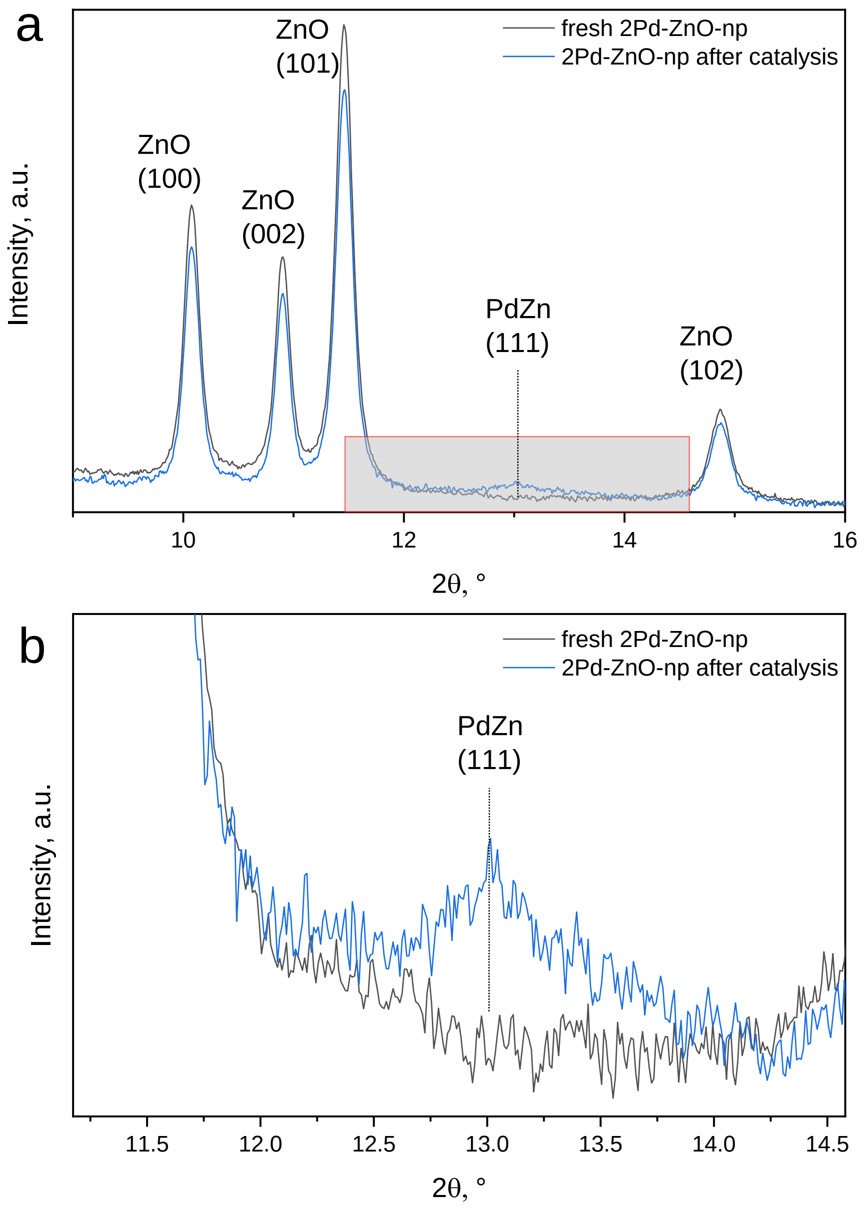

Figure S6. (a) XRD patterns of fresh 2Pd-ZnO-np catalyst (black) and spent 2Pd-ZnO-np catalyst (blue) collected during combined operando XAS-XRD study at 260 °C and 15 bar (λ= 0.4975 A). (b) shows an enlarged region of (a), where PdZn (111) diffraction peak appears after catalytic carbon dioxide hydrogenation.

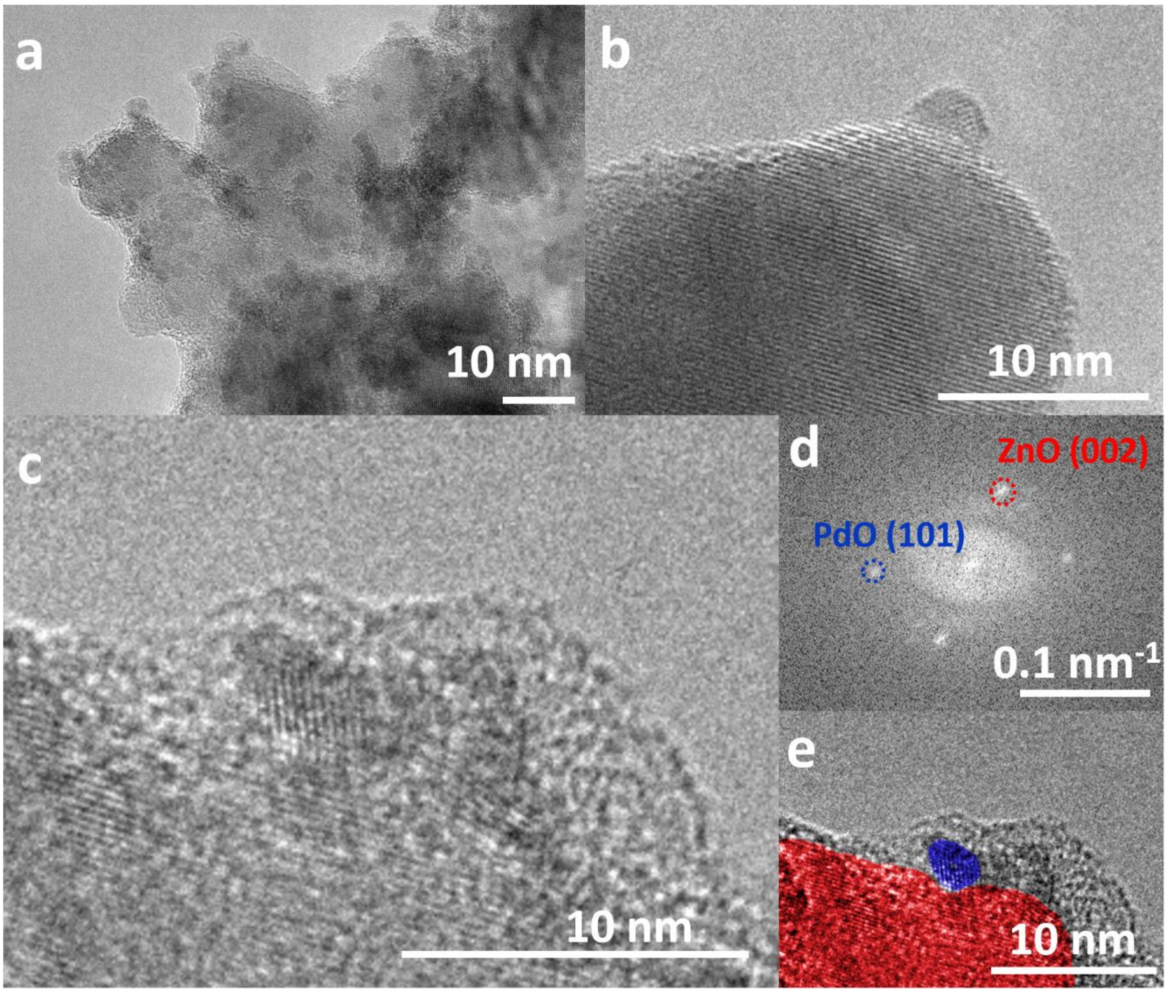

Figure S7. TEM micrographs of fresh 2Pd-ZnO-np catalyst before pre-treatment in hydrogen (a-c). Fast Fourier Transform (FFT) of (c) shows crystal lattice vectors of zinc oxide and palladium oxide (d). (e) shows superimposition of original micrograph of 2Pd-ZnO-np sample (c) with Inverse Fast Fourier Transform (IFFT) of (d) where annular masks were applied at lattice spacing of 1/2.60 Å^{-1} corresponding to (002) planes of zinc oxide (red), and at lattice spacing of 1/2.63 Å^{-1} corresponding to (101) planes of palladium oxide (blue). Although these d-values are rather similar, the darker contrast of the smaller particles indicate the presence of Pd there. Moreover, this observation is in accordance with the EDXS mappings.

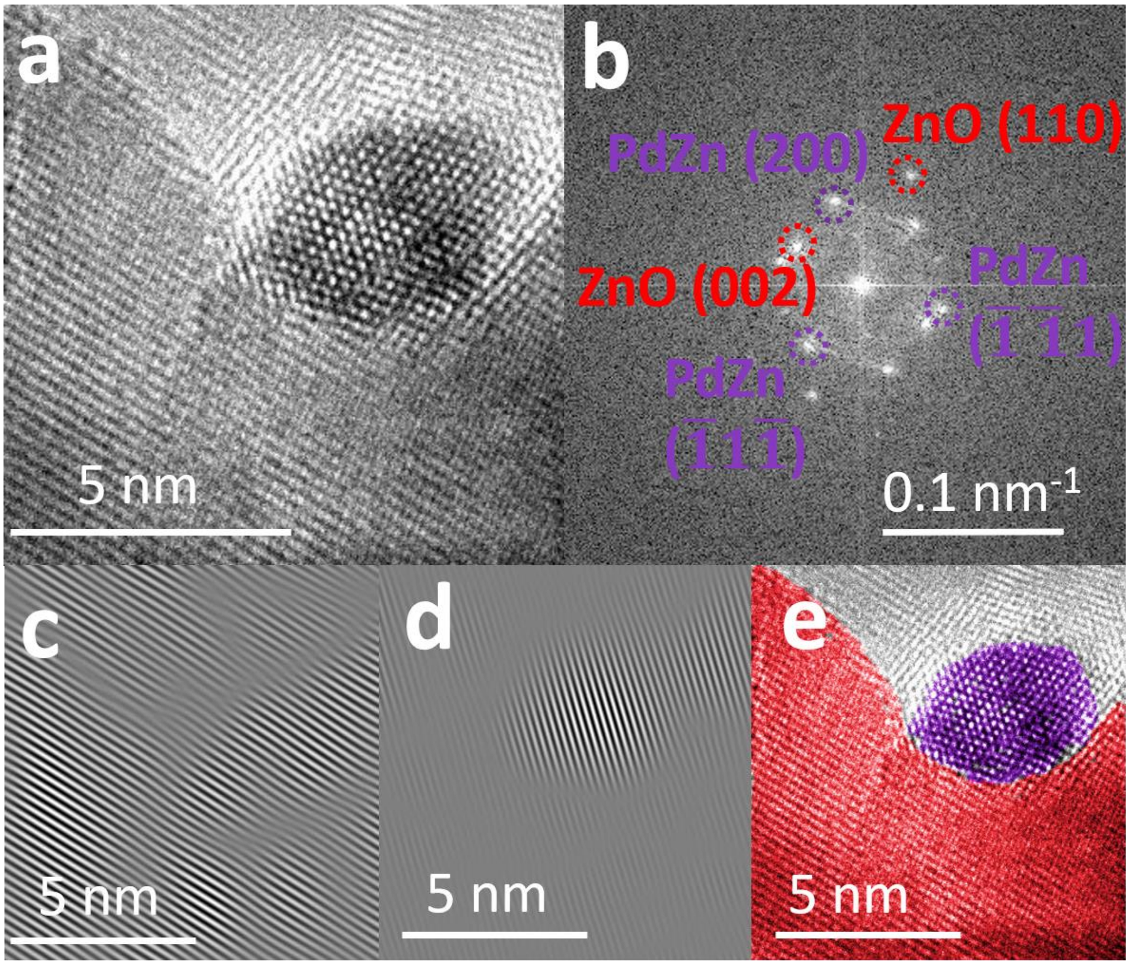

Figure S8. TEM micrographs of 2Pd-ZnO-np catalyst after catalytic carbon dioxide hydrogenation (a). Fast Fourier Transform (FFT) of (a) shows crystal lattice vectors of zinc oxide and palladium-zinc alloy (b). (c) Inverse Fast Fourier Transform (IFFT) of annular masks applied to the FFT (b) at 1/2.6 $Å^{-1}$ and 1/1.63 $Å^{-1}$ corresponding to (002) and (110) planes of zinc oxide. (d) Inverse Fast Fourier Transform (IFFT) of annular masks applied to the FFT (b) at 1/2.05 $Å^{-1}$ corresponding to (200) planes of palladium-zinc alloy. (e) The original micrograph of 2Pd-ZnO-np sample with colored crystal phase domains of zinc oxide (red) and palladium-zinc alloy (violet) superimposed.

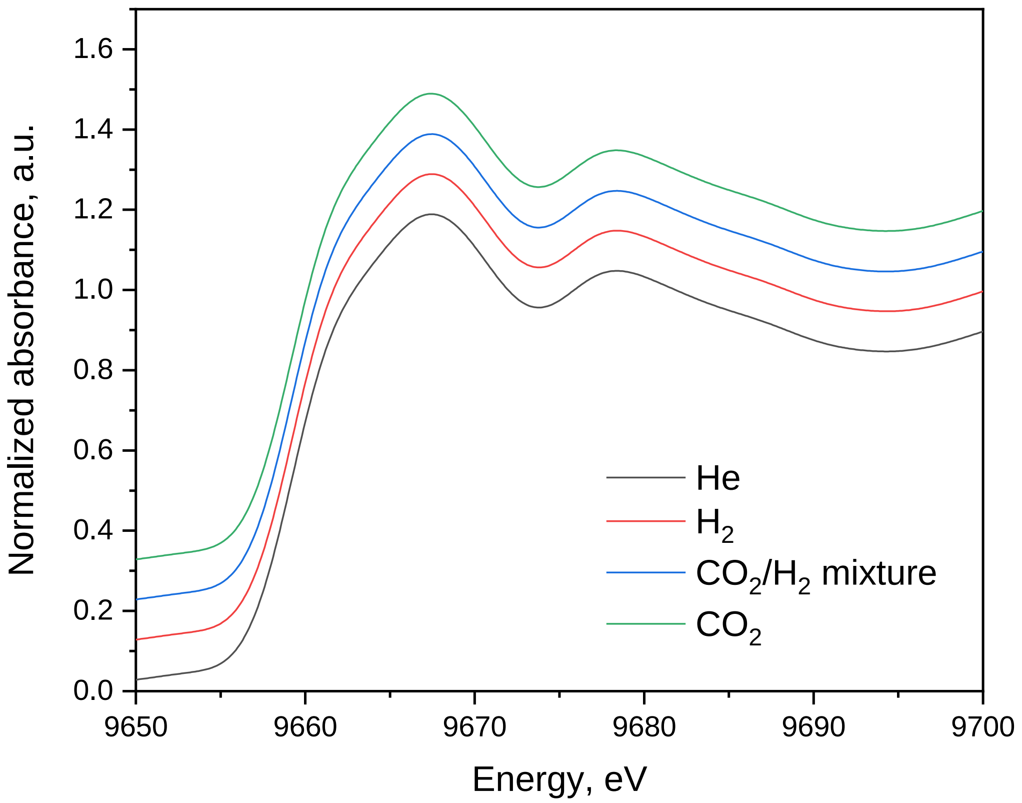

Figure S9. Zn K edge XANES spectra collected during activation and catalytic carbon dioxide hydrogenation at 260 °C and 15 bar pressure over 2Pd-ZnO-np catalyst: in helium (black), after reduction in hydrogen (red), during catalytic carbon dioxide hydrogenation (blue) and after switch to pure carbon dioxide (green);

Figure S10a shows the k^3-weighted Pd K-edge EXAFS derived from the 2Pd-ZnO-np sample obtained under four sets of conditions (as indicated): under a helium flow at 260 °C and a pressure of 15 bar; under a flow of hydrogen at 260 °C/15bar; under catalytic (CO_2/H_2) feed at 260 °C/15bar; and then, finally, under a flow of carbon dioxide again at 260 °C/15bar. Figure S10b then shows the equivalent data rendered in Fourier transform representation of the k^3-weighted EXAFS. In both cases, the black lines pertain to the experimental data, the red lines to the fits to that data derived from analysis using EXCURV.[31] Table S3 then gives the structural and statistical information arrived at analytically to yield the fits.

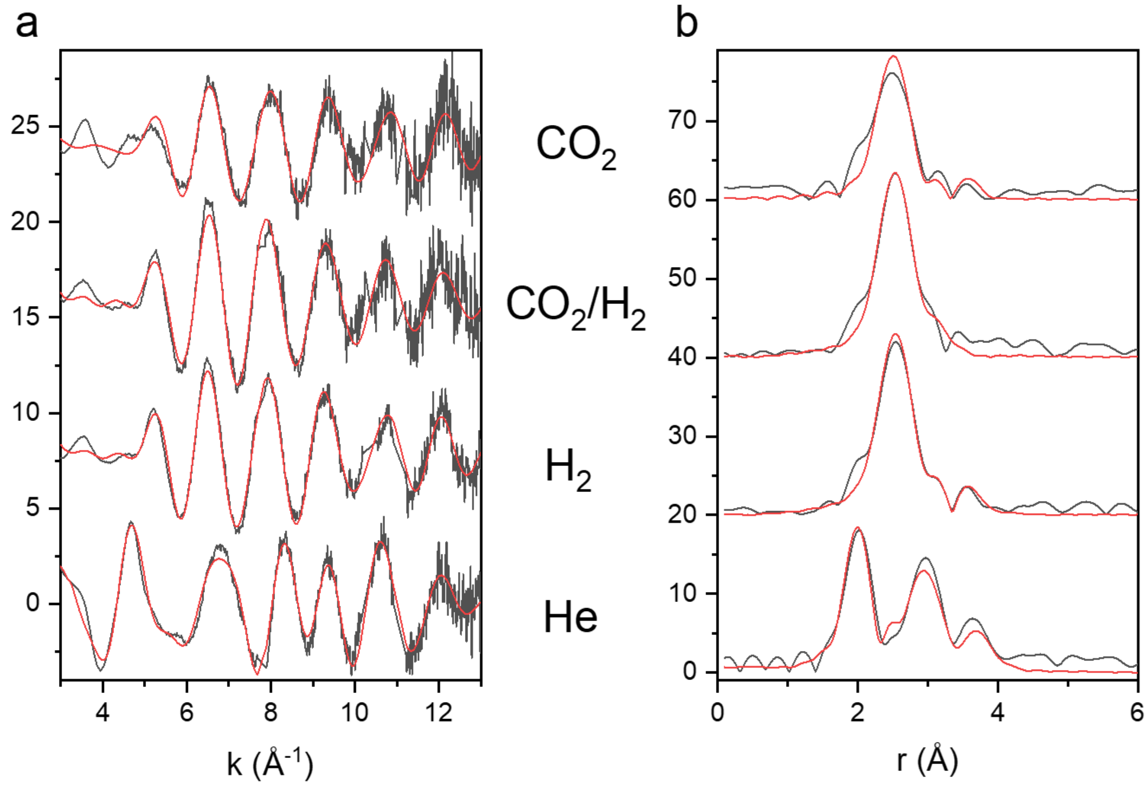

Figure S10. (a) k^3-weighted Pd K-edge EXAFS derived from the 2Pd-ZnO-np catalyst under different sets of conditions (as indicated): under a helium flow at 15 bar pressure and at 260 °C; under flowing hydrogen at 260 °C and 15 bar; at steady state under a catalytic CO_2/H_2 reaction mixture at 15 bar and 260 °C; and under a flow of carbon dioxide at 260 °C and 15 bar. (b) the corresponding, phase corrected, Fourier transform representation of the k^3-weighted EXAFS. In each case the black lines concern the experimental data, the red, fits to that data derived from analysis in EXCURV.[31]

Table S3. Structural and statistical parameters derived from the fitting of k^3-weighted Pd K-edge EXAFS for the 2Pd-ZnO-np catalyst under different conditions. K_{min}=2.5, K_{max}=13, AFAC=0.9

Conditions	Element	N[a]	r(Å)[b]	DW[c]	E_F[d]	R[e]	Chi[2f]
He	O	2.4	2.02	0.008	1.83	28.35	0.55
	Pd	2.3	2.74	0.018			
	Pd	1.2	3.03	0.016			
	Pd	2.9	3.43	0.024			
H_2	Zn	4.4	2.59	0.019	-6.1 (-6.6)	25.3 (25.7)	0.45 0.43
	Pd	2.2	2.95	0.029			
	Pd (Zn)	0.9	3.51 (3.69)	0.014			
Mix	Zn	4.4	2.58	0.019	-5.5	45.8	1.25
	Pd	2.1	2.92	0.027			
CO_2	Zn	3.1	2.57	0.018	-6.5 (-6.7)	48 (47)	1.83 0.41
	Pd	0.8	2.95	0.018			
	Pd (Zn)	0.7 (0.6)	3.47 (3.62)	0.014			

[a] N = Coordination number

[b] R = bond distance in Angstrom

[c] DW = Debye-Waller (disorder) factor ($2\sigma^2$) where σ^2 = mean squared displacement of the atom pair with respect to each other

[d] E_F = Fermi energy (eV)

[e] R% = $\Sigma_i^N 1/\sigma_i (\chi_i^e (k) - \chi_i^t (k))^2$ x 100% Where χ_i^e and χ_i^t are the experimental and theoretical EXAFS respectively, and k is the photoelectron wave vector ($Å^{-1}$). σ_i is the uncertainty in the data, with $1/\sigma_i = k_i^n / \Sigma_j^N k_i^n (\chi_i^e (\kappa_j))^2$..

[f] Chi^2 = statistical measure of goodness of fit (x 10^{-6})

Consistent with the previously shown XANES, EXAFS yields a picture of 2Pd-ZnO-np sample under 15 bar He at 260 °C as being predominantly oxidized in nature, and with two Pd-Pd scattering shells, at ca. 3.0 and 3.4 Å respectively, which are indicative of a nanoscale, PdO structure. However, alongside this, the EXAFS indicates the presence of a minority, metallic Pd component (an additional Pd-Pd shell at ca. 2.74Å). In other words, a low level of reduced Pd is either inherent to this sample, or has been created by heating in 15 bar He to 260 °C.

A switch to a hydrogen flow precipitates a complete reduction of the Pd present, along with some of the ZnO, to yield a highly dispersed PdZn alloy phase consistent with an $L1_o$ structure

(R_{Pd-Pd} = 2.9 Å, R_{Pd-Zn} = 2.6 Å) [22]. A further scattering interaction may be fitted to either Pd (3.51 Å) or Zn (3.69 Å) though, as can be seen from Table S3, it is not formally, by either change in R factor or Chi^2, significant. The highly dispersed nature of this phase is indicated by both the reduced levels of coordination in both Pd-Zn and Pd-Pd shells compared to the bulk case (N_{PdZn} = 8, N_{PdPd} = 4 [22]) and the lack of significant higher shell structure. In this sense, the EXAFS is therefore consistent with the XRD (Figure 2e), which reveals only very broad and weak reflections that may be associated with this phase.

A switch to the catalytic reaction feed does not elicit in any significant changes in the EXAFS and, overall, the k^3- weighted EXAFS envelope remains rather similar to that observed under pure hydrogen. As such, once again, the alloy nature of the supported phase does not change radically as a result in the change of feed, save for in this case, there is no evidence in the FT for the presence of a third shell at around 3.5 Å.

A switch to a carbon dioxide flow, however, elicits a substantial change in the k^3-weighted EXAFS, which principally manifests itself in a significant reduction in amplitudes. In Table S3 this is reflected in a net decrease in the apparent coordination numbers associated with the Pd-Zn (2.57 Å) and Pd-Pd ((2.95 Å) interactions. As before, the third shell may also be fitted to either Pd or Zn but, again, with the recognition that, in terms of the goodness of fit obtained to the k^3-weighted EXAFS through the addition of this shell, it cannot be regarded as significant.

Exposure to carbon dioxide at 15 bar and 260 °C does not seem to have affected the PdZn alloy nature of the sample, but has elicited some other sort of change, such as a decreased in average particle size, change in morphology, or overall levels of static disorder. However, whilst the results, especially for the Pd-Pd interaction, might indicate a substantial change in this respect, the correlation that exists between coordination numbers in EXAFS and the Debye-Waller (DW) term, does not permit us to specify with any certitude the source of this change. Examination of the correlations that exists between the Pd-Pd coordination number and the associated DW term, show that the former may exist anywhere in the range 0.5 -2 and the latter, between 0.01 and 0.028 for virtually no change to the R factor or Chi^2 parameter. As a result, therefore, whilst the EXAFS confirms the retention of the PdZn alloy motif, it does not permit us to specify the effect of the carbon dioxide on the nanoparticles present in any more precise way than to state that the presence of this gas leads to a significant (static) disordering of this phase.

Overall therefore, in this case, Pd K-edge EXAFS shows that, once reduced in hydrogen, the nanoparticulate PdZn phase is retained under the conditions applied, irrespective of the gaseous

environment it experiences. Whilst some evidence is forthcoming to indicate that this supported phase is, in some way subject to heightened levels of static disorder in the presence of pure carbon dioxide, any further specification as to how these increased levels of disorder manifest themselves on the nanoscale is not possible on the basis of this data.

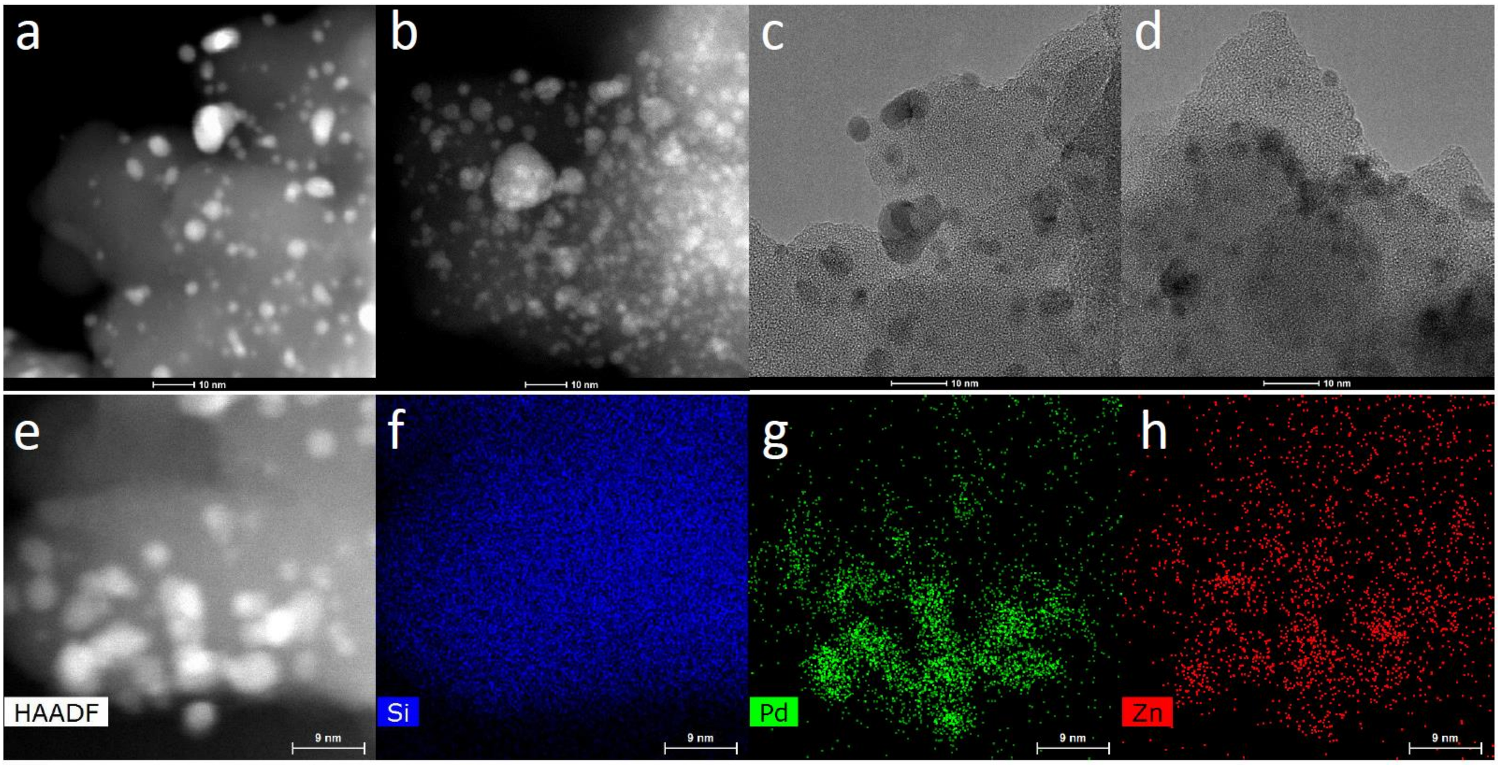

Figure S11. STEM micrographs (a-b), TEM (c-d) as well as STEM (e)combined with energy dispersive X-ray elemental mapping (f-h) of PdZn/SiO$_2$ catalyst after catalytic carbon dioxide hydrogenation at 260 °C and 30 bar pressure.

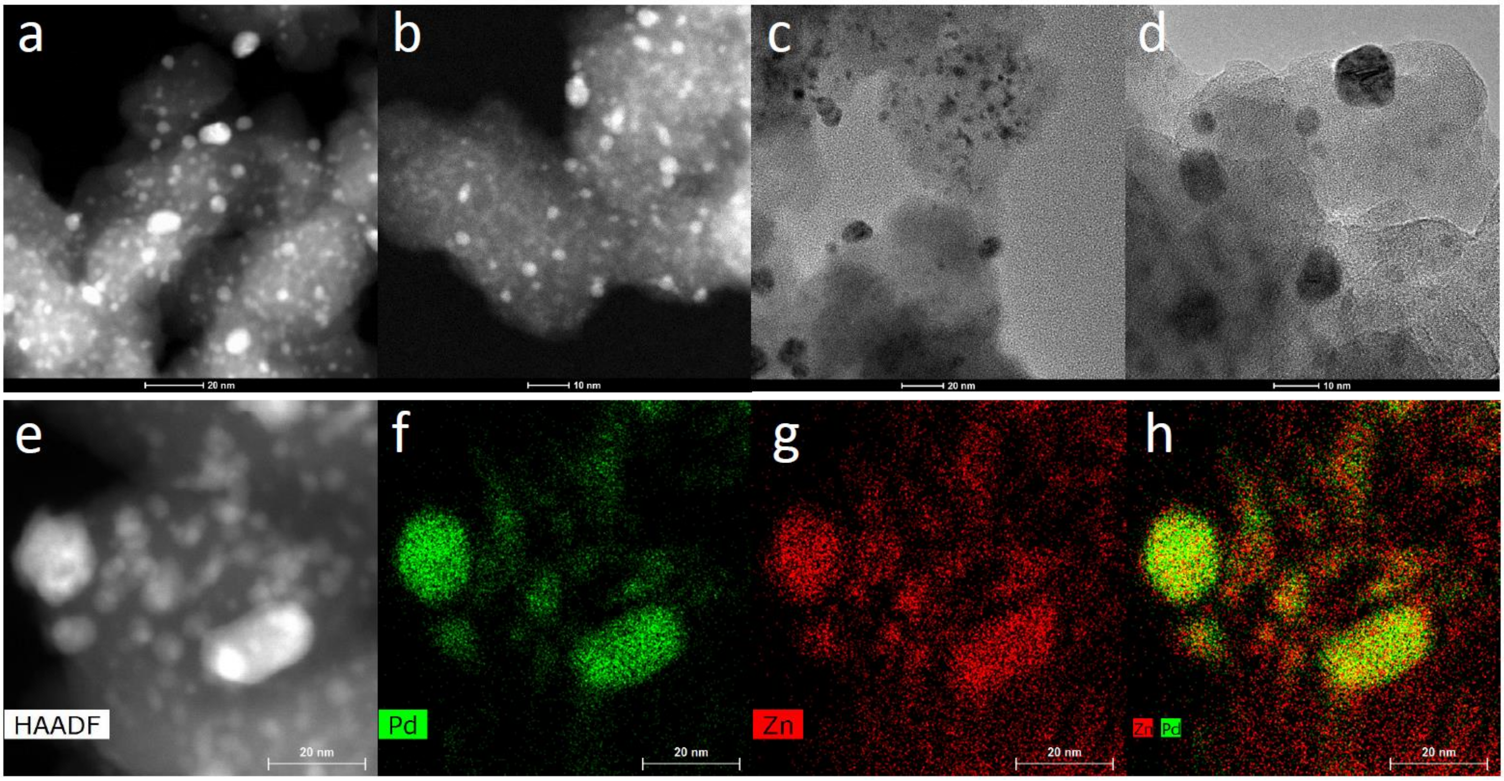

Figure S12. STEM micrographs (a-b), TEM (c-d) as well as STEM (e)combined with energy dispersive X-ray elemental mapping (f-h) of PdZn/ZnO/SiO$_2$ catalyst after catalytic carbon dioxide hydrogenation at 260 °C and 30 bar pressure.

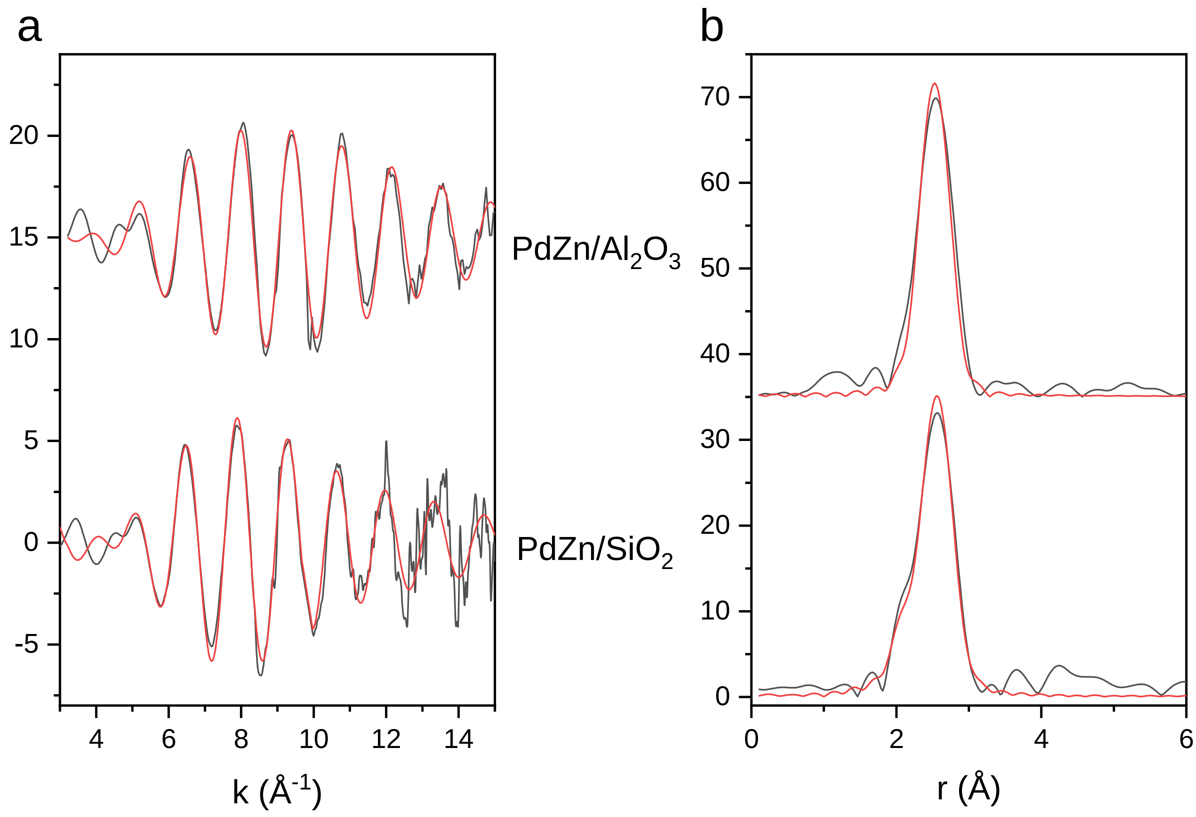

Figure S13. (a) k^{3}*-weighted Pd K-edge EXAFS derived from (as indicated) PdZn/SiO*$_2$ *and PdZn/Al*$_2$*O*$_3$ *catalysts post reduction in hydrogen; (b) the corresponding Fourier transforms of the k*^{3}*-weighted data. In each case, the black lines refer to the experimental data, the red, to fits to that data resulting from analysis using EXCURV* [31].

Figure S13a shows the k^{3}-weighted Pd K-edge EXAFS derived from (as indicated) PdZn/SiO$_2$ and PdZn/Al$_2$O$_3$ catalysts post reduction in hydrogen. Figure S13b shows the corresponding Fourier transforms of the k^{3}-weighted data. In each case, the black lines refer to the experimental data, the red, to fits to that data resulting from analysis using EXCURV.[31] Table S4 then gives the structural and statistical data derived from the analysis.

Both of these systems are characterized by a Pd-Zn scattering shell at ca. 2.57 – 2.6 Å. As such, and as previously, the formation of a nano-sized PdZn alloy is indicated. In contrast to the 2Pd-ZnO-np cases previously shown (Figure S10), however, no significant PdPd shell ca. 2.95 Å is evidenced. Such a shell can be included and fitted to the data, but the addition of such a shell makes no difference to the goodness of fit and the DW factors associated with co-ordinations of only unity or less are unusually high ($\approx$ 0.035). As such, if present at all in these materials this second shell of coordination is highly disordered.

Table S4. Structural and statistical parameters derived from the fitting of k^3-weighted Pd K-edge EXAFS for the PdZn/SiO_2 and PdZn/Al_2O_3 catalysts after pre-treatment in hydrogen. K_{min}=2.5, K_{max}=15, AFAC=0.9

Sample	Element	N[a]	r(Å)[b]	DW[c]	E_F[d]	R[e]	Chi[2f]
PdZn/SiO_2	O	0.7	2.16	0.004	-9.1	33.2	0.84
	Zn	4.0	2.60	0.013			
PdZn/Al_2O_3	Zn	3.0	2.57	0.010	-1.3	22.4	0.5

[a] N = Coordination number

[b] R = bond distance in Angstrom

[c] DW = Debye-Waller (disorder) factor ($2\sigma^2$) where σ^2 = mean squared displacement of the atom pair with respect to each other

[d] E_F = Fermi energy (eV)

[e] R% = $\Sigma_i^N 1/\sigma_i (\chi_i^e(k) - \chi_i^t(k))^2 \times 100\%$ Where χ_i^e and χ_i^t are the experimental and theoretical EXAFS respectively, and k is the photoelectron wave vector (Å^{-1}). s_i is the uncertainty in the data, with $1/\sigma_i = k_i^n / \Sigma_j^N k_i^n (\chi_i^e(k_j))^2$.

[f] Chi^2 = statistical measure of goodness of fit (x 10^{-6})

However, there are also significant differences between the two systems. Firstly, whilst the amplitudes of the EXAFS are similar in each case, a measurable phase shift between these two cases can be observed across the range of the k^3-weighted EXAFS, with the EXAFS due to the PdZn/Al_2O_3 system being shifted to higher k(Å^{-1}). This is indicative of a contraction of the average Pd-Zn bond distance in this sample compared to the PdZn/SiO_2 case. Analysis shows that the magnitude of the contraction is small (2.57 versus 2.6 Å i.e. 0.03 Å) but it is, nonetheless, indicated to be present. Secondly, as evidenced from the Fourier transforms of the EXAFS (Figure S13b), there is a pronounced difference between the two cases in terms of higher shell structure, with the PdZn/SiO_2 yielding evidence above 3 Å in the FT of a developed higher shell environment whereas this is, to all intents, absent in the PdZn/Al_2O_3 case. Lastly, in the PdZn/SiO_2 case, a low level of O coordination (2.16 Å) can also be fitted, whereas it is absent in the PdZn/Al_2O_3 system. In the former case, the addition of this shell decreases the R factor and Chi^2 by ca. 10% and therefore can be considered as significant, whereas in the latter the addition of such a shell marginal at best in terms of the quality of the fit achieved.

As such, the Pd K-edge EXAFS in both of these systems confirms for the formation of PdZn alloys but, at the same time, shows that in a number of ways these phases are subtly different from each other according to the oxide upon which they are supported. To a first approximation, the observance of a defined higher shell structure and longer Pd-Zn first shell

bond distances in the PdZn/SiO_2 case would indicate that the alloys formed in this case are significantly more ordered, and possibly larger, than in PdZn/Al_2O_3. The optimally refined values for the first Pd-Zn scattering interaction (4 versus 3) would also be consistent with this proposition. However, and as with the previous Pd K-edge EXAFS analysis for the 2Pd-ZnO-np case, the correlations that exist between the coordination number and the DW factors mean that we cannot be certain of this difference.

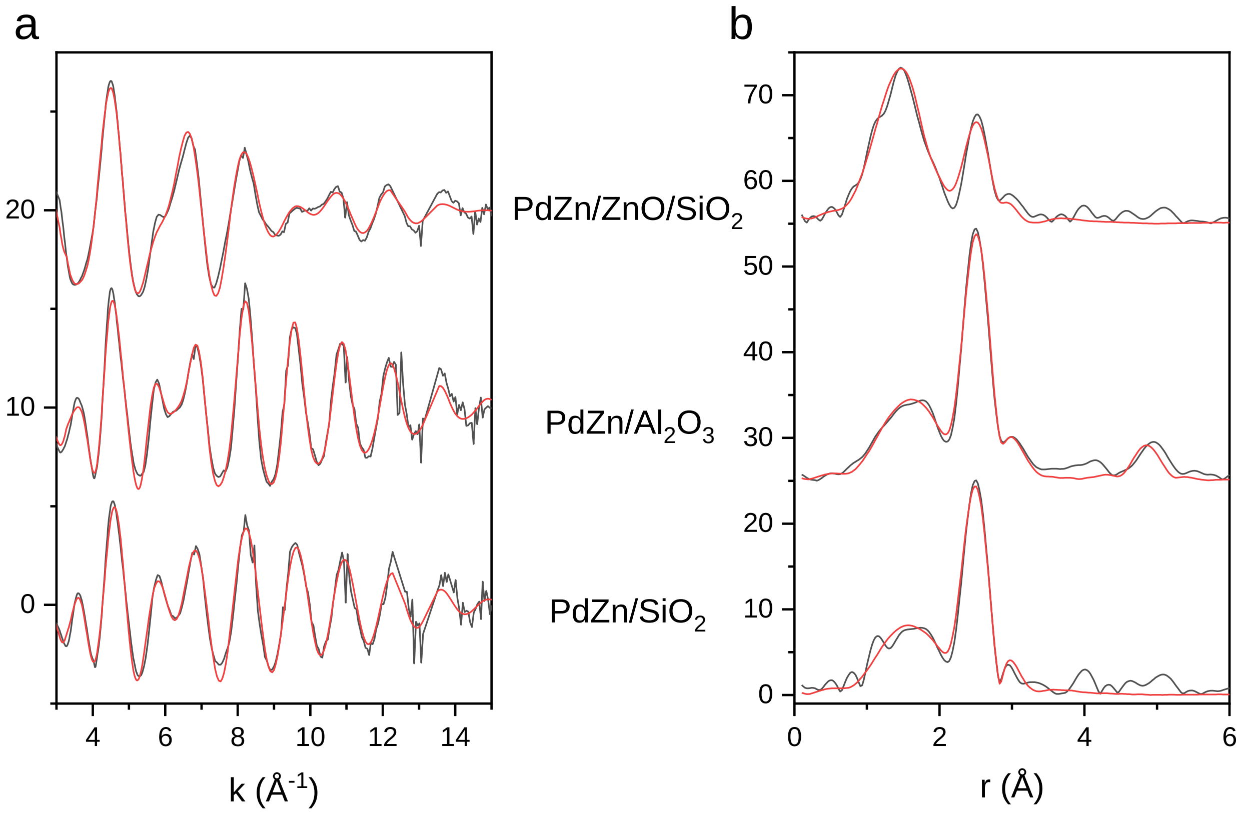

Figure S14. (a) k^3-weighted Zn K-edge EXAFS derived from (as indicated) PdZn/SiO_2, PdZn/Al_2O_3 and PdZn/ZnO/SiO_2 catalysts after catalytic carbon dioxide hydrogenation at 260 °C and 30 bar pressure; (b) the corresponding Fourier transforms of the k^3-weighted data. In each case, the black lines refer to the experimental data, the red, to fits to that data resulting from analysis using EXCURV.[31]

The overall Zn K-edge EXAFS scattering envelope (Figure S14a) for both PdZn/SiO_2, PdZn/Al_2O_3 samples appears very similar, whereas that derived from the PdZn/ZnO/SiO_2 is radically different beyond ca. k = 5 $Å^{-1}$. The source of this difference is shown graphically by the FT representation of the k^3-weighted EXAFS (Figure S14b). For PdZn/SiO_2, PdZn/Al_2O_3 the phase-corrected FTs are dominated by the scattering shell at ca. 2.6 Å that we may associate (see also Table S5) with the significant presence of the PdZn alloy phase. In these two cases, this is accompanied by a very broad feature in the FT, indicative of low Z (modelled as O) coordination to some fraction of the Zn. The breadth of this feature might indicate that a high

level of disorder is associated with this interaction, and that most likely a number of different Zn-O bonding motifs are present post catalysis. Given that the XANES, from these two cases (Figure 3d), points squarely to a predominance of zinc being present in a reduced form, and such low Z coordination is not evidenced at all to any significant degree in the Pd K-edge spectra (Figure 3c), this rather significant level of coordination might suggest a significant segregation of the zinc toward the surface of the alloy nanoparticles particles where they may be in contact with either reactant species or the underlying support. Furthermore, whilst very similar to each other, in these senses, the FTs and subsequent analysis, show that in others these two systems are rather different in terms of the longer range levels of order that are indicated to be present within them.

In the PdZn/SiO_2 case, whilst there is some evidence for scattering shells beyond ca. 3.5 Å, this structure, is relatively weak and not so significant in terms of the total FT envelope. By contrast, in PdZn/Al_2O_3 this higher shell structure is far more developed, and specifically for the scattering feature at ca. 5 Å. As shown in the fits, and in Table S5, this feature can be well-fitted assuming a further Zn-Pd scattering interaction @ ca 4.9 Å. Moreover, the addition of this shell is highly significant in terms of the effects it has on both the R factor and Chi^2, which drop from 25 to 19 and 0.76 to 0.43 (x 10^{-6}) concurrent with its addition. However, this feature may also be accounted for to some degree by multiple scattering effects that arise from scattering interaction across a two-dimensional (d2h) arrangement of 4 Pd atoms around a central Zn atom. Overall, therefore, the Zn K-edge EXAFS demonstrates that whilst the PdZn phases present in these two samples are nominally similar, this phase, when formed on Al_2O_3, is significantly more ordered, in at least two dimensions, that its counterpart supported upon SiO_2.

Different from both of these cases is the PdZn/ZnO/SiO_2. Consistent with the reduced intensity of the low binding energy pre-edge feature observed in XANES, the EXAFS is dominated by a broad low Z (O) scattering shell (Figure 3f). Above ca. 3 Å there is also precious little evidence of any well-defined higher shells of coordination. In between these extremes, however, we find the shell that is indicative of the presence of the PdZn alloy, but at a much reduced level in terms of its contribution to the overall EXAFS envelope. Together with the XANES (Figure 3d) this sample is therefore indicated to be comprised of both PdZn alloy and zinc oxide phases with a much heavier weighting to the latter than the former. In contrast, in the PdZn/SiO_2 and PdZn/Al_2O_3 cases, it is evident from the XANES and the EXAFS that considerably more of the zinc in the system is present in a metallic form within supported PdZn nanoparticles.

Lastly we note that (vide infra) the Zn-Zn scattering shell found to be present in all of these samples post reaction at ca. 2.9 – 3 Å could, hypothetically, also be the result of the Zn-Pd interaction expected upon the basis of the PdZn alloy bulk structure (r = 2.95 Å). Attempts to fit this interaction using a Pd scatterer, rather than Zn, fail in the sense that they return unrealistically short bond distances coupled to excessively large DW factors. As such, this Zn-Zn scattering interaction we associate with some form of disordered ZnO phase, the levels of which vary greatly between the three cases but which are greatest in the PdZn/ZnO/SiO_2 sample. Once again, we further note that, in this case in particular, the correlations between N and DW factor mean that little precision can be attached to the values of N, which in each case could realistically range between 1 and 4.

Table S5. Structural and statistical parameters derived from the fitting of k^3-weighted Zn K-edge EXAFS for the PdZn/SiO_2, PdZn/Al_2O_3 and PdZn/ZnO/SiO_2 catalysts after catalytic carbon dioxide hydrogenation. K_{min}=2.5, K_{max}=15, AFAC=0.9

System	Element	N[a]	r(Å)[b]	DW[c]	E_F[d]	R[e]	Chi^2[f]
PdZn/SiO_2	O	1.6	1.97	0.019	-5.5	20.6	0.60
	Pd	4.6	2.58	0.022			
	Zn†	1.2	2.95	0.033			
PdZn/Al_2O_3	O	1.5	1.95	0.017	-1.5	18.9 (25)	0.43 (0.76)
	Pd	4.9	2.58	0.021			
	Zn†	2.8	2.99	0.037			
	Pd*	4.1	4.89	0.024			
PdZn/ZnO/SiO_2	O	3.6	1.95	0.017	-2.2	15.1	0.49
	Pd	2.4	2.59	0.024			
	Zn†	3.5	2.91	0.039			

† Exchanging Zn for Pd in this shell fails, as such, this is not due to the expected Pd shell from a Pd Zn alloy phase @ 2.95 Å.

*Single scattering analysis only: this scattering interaction can also be described to an extent solely upon the basis of multiple scattering occurring across a $ZnPd_4O_2$ unit of d_2h symmetry. R and Chi^2 values in brackets refer to the goodness of fit obtained in the absence of the Pd shell @ 4.89 Å, or the use of full curved wave multiple scattering calculations.

[a] N = coordination number.

[b] R = bond distance in Angstrom.

[c] DW = Debye-Waller (disorder) factor ($2\sigma^2$) where σ^2 = mean squared displacement of the atom pair with respect to each other.

[d] E_F = Fermi energy (eV)

[e] R% = $\Sigma_i^N 1/\sigma_i (\chi_i^e(k) - \chi_i^t(k))^2$ x 100% Where χ_i^e and χ_i^t are the experimental and theoretical EXAFS. respectively, and k is the photoelectron wave vector ($Å^{-1}$). s_i is the uncertainty in the data, with $1/\sigma_i = k_i^n / \Sigma_j^N k_i^n (\chi_i^e(k_j))^2$.

[f] Chi^2 = statistical measure of goodness of fit (x 10^{-6})
